# A review of the genus *Angilia* Stål, 1865 (Hemiptera: Heteroptera: Veliidae): New species, new synonyms, checklist, and key to species

**DOI:** 10.1371/journal.pone.0332848

**Published:** 2025-09-29

**Authors:** Higor D. D. Rodrigues, Robert W. Sites, Felipe F. F. Moreira

**Affiliations:** 1 Laboratório de Entomologia, Instituto Oswaldo Cruz, Fundação Oswaldo Cruz, Rio de Janeiro, Rio de Janeiro, Brazil; 2 Enns Entomology Museum, Division of Plant Sciences, University of Missouri, Columbia, Missouri, United States of America; Universidade Estadual Paulista: Universidade Estadual Paulista Julio de Mesquita Filho, BRAZIL

## Abstract

*Angilia* Stål, 1865 is one of three genera of the subfamily Veliinae (Hemiptera: Heteroptera: Veliidae) from the Eastern Hemisphere and is distributed in sub-Saharan Africa, Madagascar, and Southeast Asia. Currently, 23 species and five subspecies are known and the genus is divided into two subgenera: *Angilia* (*Angilia*) Stål, 1865 and *A*. (*Adriennella*) Poisson, 1942. We present here the descriptions of two new species from Africa: *A*. (*An.*) *morogoro* **n. sp.**, from Tanzania, and *A.* (*Ad.*) *igniventris* **n. sp.**, from Madagascar. In addition, we propose *A.* (*An.*) *albidotincta ugandensis* Poisson, 1955 as a junior subjective synonym of *A.* (*An.*) *a. albidotincta* (Stål, 1855); *A.* (*An.*) *kaokoveldi* Poisson, 1958a as a junior objective synonym and junior homonym of *A*. (*An*.) *kaokoveldi* Poisson, 1957; *A*. (*An*.) *albidotincta kaokoveldi* Poisson, 1958b as a junior subjective synonym and junior homonym of *A*. (*An*.) *kaokoveldi* Poisson, 1957; *A*. (*An*.) *collarti* Poisson, 1958a as a junior objective synonym and junior homonym of *A*. (*An*.) *collarti* Poisson, 1957; transfer *Angilia perplexa* Poisson, 1942 from the subgenus *Adriennella* to *Angilia s. str*.; and provide morphological notes and illustrations for the following species: *A*. (*An*.) *aeterna* Hoberlandt, 1946, *A.* (*An.*) *albidotincta*, *A.* (*An.*) *ambakakae* Poisson, 1952, *A.* (*An.*) *congoensis* Poisson, 1950, *A*. (*An*.) *kaokoveldi*, *A.* (*An.*) *rhodesiensis* Poisson, 1955, *A.* (*Ad.*) *anderseni* Zettel & Hecher, 1998, *A.* (*Ad.*) *bertrandi* Poisson, 1963, *A.* (*Ad.*) *bispinosa* Andersen, 1981, *A.* (*Ad.*) *conradsi* Poisson, 1950, *A.* (*Ad.*) *orientalis* Andersen, 1981, *A.* (*Ad.*) *philippiensis* Drake & Hoberlandt, 1953, and *A.* (*Ad.*) *schoutedeni schoutedeni* Poisson, 1942. We also designate lectotypes for three species and present a checklist and key to the species.

## Introduction

The subfamily Veliinae (Hemiptera: Heteroptera: Veliidae) is represented in the Eastern Hemisphere by three genera: *Angilia* Stål, 1865, *Angilovelia* Andersen, 1981, and *Velia* Latreille, 1804 [1]. *Angilia* occurs in sub-Saharan Africa, Madagascar, and Southeast Asia, and is currently divided into two subgenera: *Angilia* (*Angilia*) Stål, 1865 and *A*. (*Adriennella*) Poisson, 1942. It contains 23 species and five subspecies [2,3].

*Angilia* (*s. str*.) comprises 15 taxa (12 species and three subspecies) and is restricted to sub-Saharan Africa and Madagascar. It is characterized by antennomeres III and IV about as thick as antennomere II; the pronotum moderately elevated between the humeral angles and without a finger-like projection on the posterior margin; the grasping comb of the foretibia clearly shorter in females than in males; and the middle tarsus subequal in length to the middle tibia [2]. The subgenus *A*. (*Adriennella*) contains 13 taxa (11 species and two subspecies) and has a wider distribution, occurring in the same areas as the nominal subgenus and also in Southeast Asia. It is characterized by antennomeres III and IV distinctly thinner than antennomere II; the pronotum strongly elevated between the humeral angles and with a finger-like projection on the posterior margin; the grasping comb of the female foretibia almost as long as that of the male; and the middle tarsus about 2/3 as long as the middle tibia [2,3].

Raymond A. Poisson, one of the most prolific specialists on African Veliidae, described 17 of the 21 taxa recorded from the continent [4–10]. His descriptions are usually accompanied by illustrations, but they do not always facilitate identification, forcing the reader to make comparisons with several other descriptions. This fact, together with the lack of keys, makes it difficult to identify specimens from Africa. The opposite is true for the Asian fauna, for which original descriptions are more detailed, and there are informative drawings and an identification key [2,3,11].

Zettel & Hecher [3] divided the Asian fauna into two species groups: *A. orientalis* group and *A. bispinosa* group. The *A. orientalis* group is identified by the following characteristics: pronotal humeri without spines, a slender hind femur with short ventral spines, a very large abdominal segment VIII in males, and a broad paramere with a rounded or truncated apex. In contrast, species in the *A. bispinosa* group have spines on the pronotal humeri, a distinctly thickened hind femur set with distinct spines ventrally, and a male with a moderate-sized abdominal segment VIII, and a thin falciform paramere (male unknown for *A. borneensis* and *A. trispinosa*) [3].

In the present study, after examining material from mainland Africa, Madagascar, and Southeast Asia, we describe two new species of *Angilia*. Additionally, we propose synonymies, homonymies, and a subgeneric transference, and present morphological notes, lectotype designations, new records, a checklist, and a key to the species assigned to the genus.

## Materials and methods

Material examined is organized in alphabetical order according to the country and internal political divisions. A slash (/) separates data on different labels. Information given between brackets [] does not appear on the labels but was provided in the literature or inferred subsequently. All measurements are given in millimeters. Photographs were edited in Adobe Photoshop CS6. Distribution maps were created using Quantum GIS, with localities obtained from specimen labels and publications. The shapefile maps are under open access license free to use, credited to: Natural Earth database (https://www.naturalearthdata.com/), which is in the public domain and freely available for use, modification, and reproduction for educational, scientific, and commercial purposes. Photographs of collection sites identified as “L-numbers” are available in a Locality Image Database of the Enns Entomology Museum, University of Missouri. Specimens examined are deposited in the following institutions:

- CEIOC Coleção Entomológica do Instituto Oswaldo Cruz, Fundação Oswaldo Cruz, Rio de Janeiro, Brazil
- MZLU Biologiska museet, Lunds universitet, Lund, Sweden
- NHRS Naturhistoriska riksmuseet, Stockholm, Sweden
- NMNH National Museum of Natural History, Smithsonian Institution, Washington D.C., United States
- NMPC Národní muzeum, Prague, Czech Republic
- RMCA Musée royal de l’Afrique centrale, Tervuren, Belgium
- UMC University of Missouri, Columbia, United States

### Nomenclatural acts

The electronic edition of this article conforms to the requirements of the amended International Code of Zoological Nomenclature, and hence the new names contained herein are available under that Code from the electronic edition of this article. This published work and the nomenclatural acts it contains have been registered in ZooBank, the online registration system for the ICZN. The ZooBank LSIDs (Life Science Identifiers) can be resolved and the associated information viewed through any standard web browser by appending the LSID to the prefix “http://zoobank.org/”. The LSID for this publication is: urn:lsid:zoobank.org:pub:99303EF0-3847-4586-99DA-FB46D5476F4E. The electronic edition of this work was published in a journal with an ISSN, and has been archived and is available from the following digital repositories: LOCKSS.

## Results and discussion

### Taxonomy

#### *Angilia* Stål, 1865

*Angilia* Stål, 1865 [12]: 167–168 (original description).

Type species by monotypy: *Velia albidotincta* Stål, 1855: 46 (original description).

**Fig 1:**
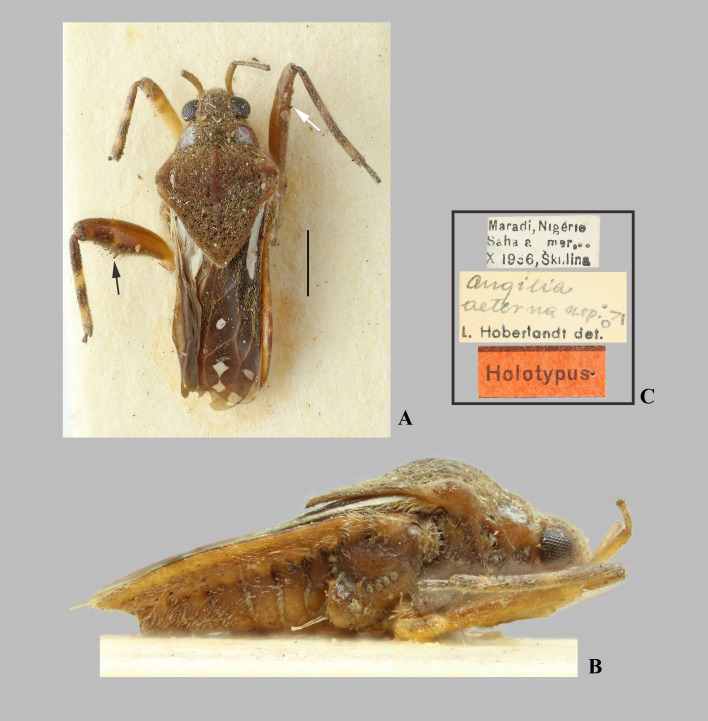
*Angilia* (*Angilia*) *aeterna* Hoberlandt, 1946. (A) dorsal and (B) lateral habitus of macropterous male holotype (NMPC), white arrow indicates large spine of middle femur, black arrow indicates large spines of hind femur; (C) holotype labels. Photographed by P. Kment (© 2025 Národní muzeum). Made available by the Národní muzeum under Creative Commons Attribution 4.0 International Public License, CC-BY 4.0, https://creativecommons.org/licenses/by/4.0/legalcode. Scale bar = 1.0 mm and applies to Fig A.

**Fig 2:**
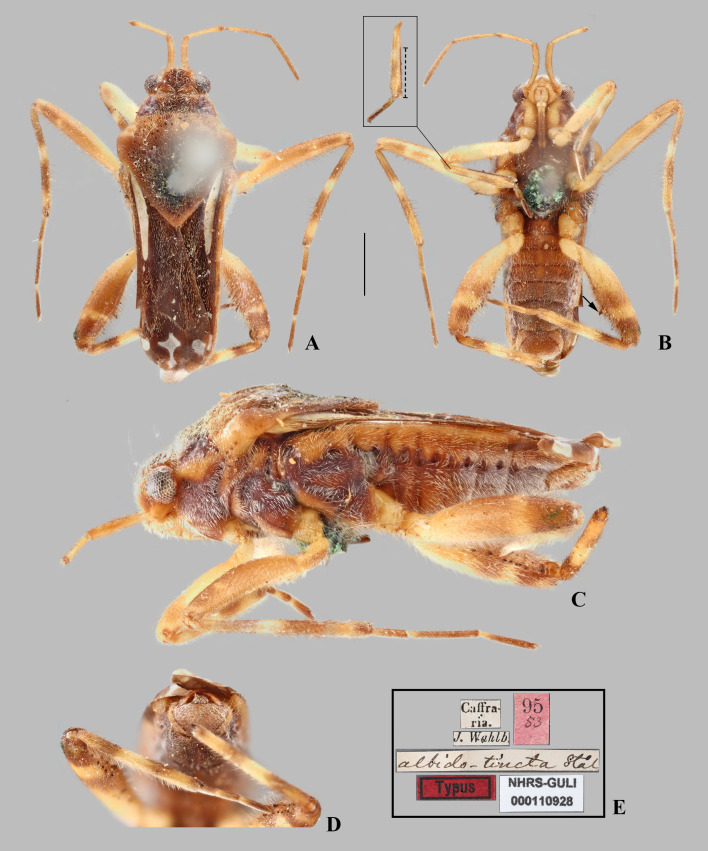
*Angilia* (*Angilia*) *albidotincta* (Stål, 1855). (A) dorsal, (B) ventral and (C) lateral habitus of macropterous male lectotype (NHRS), inset shows part of foreleg, dashed line indicates length of tibial grasping comb, black arrow indicates large spine of hind femur; (D) abdominal apex in posterior view; (E) lectotype labels. Photographed by G. Lindberg (© 2025 Naturhistoriska riksmuseet). Made available by the Naturhistoriska riksmuseet under Creative Commons Attribution 4.0 International Public License, CC-BY 4.0, https://creativecommons.org/licenses/by/4.0/legalcode. Scale bar = 1.0 mm and applies to Figs A–B.

**Diagnosis.** *Angilia* is distinguished from other Veliinae by the banded legs, the middle tarsus at least 2/3 as long as the middle tibia, and the unique maculation pattern on the forewing with a basal, a medial (sometimes absent), and a set of apical maculae (Figs 1–2).

**Fig 3:**
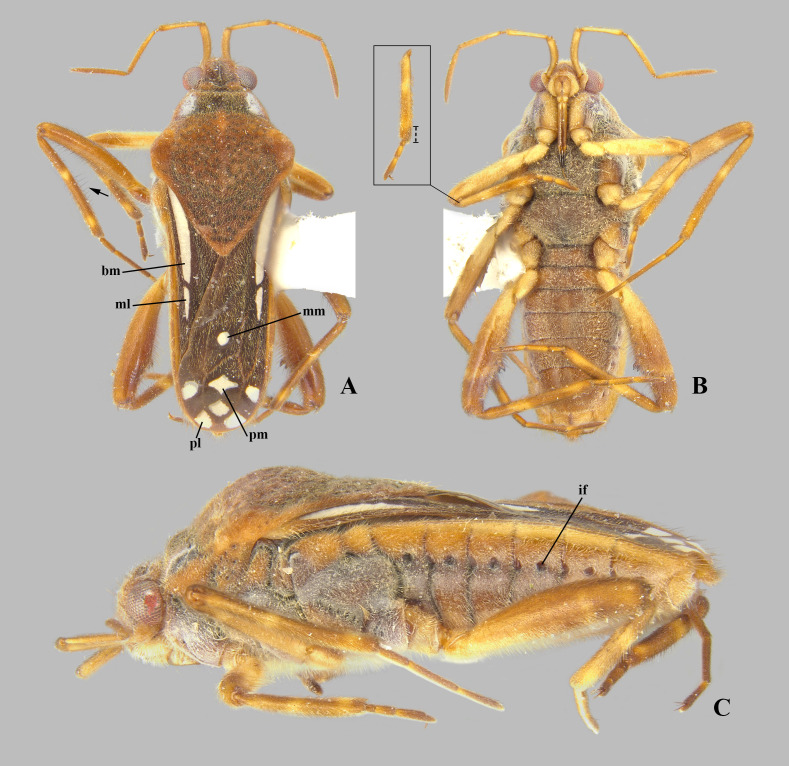
*Angilia* (*Angilia*) *albidotincta* (Stål, 1855). (A) dorsal, (B) ventral and (C) lateral habitus of macropterous female from Tanzania (UMC), inset shows part of foreleg, dashed line indicates length of tibial grasping comb, black arrow indicates elongate dark-brown trichobothria-like setae of middle tibia. bm = basal macula, if = impressed furrow, ml = mid-lateral macula, mm = medial macula, pl = posterolateral macula, pm = posteromedial macula.

**Fig 4:**
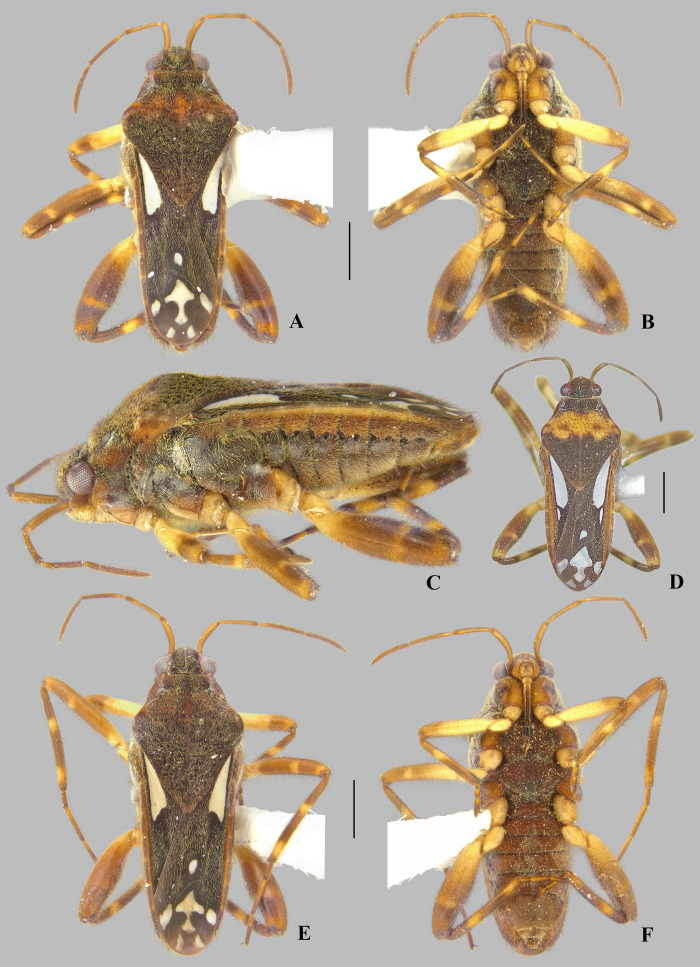
*Angilia* (*Angilia*) *ambakakae* Poisson, 1952. (A) dorsal, (B) ventral, (C) lateral habitus of macropterous male from Sava (UMC); (D) dorsal habitus of macropterous female from Menabe (NMNH); (E) dorsal and (F) ventral habitus of macropterous female from Sava (UMC). Scale bars = 1.00 mm and apply to Figs A–B, D–F.

**Supplemental description.** Andersen [2] offered a redescription of the genus, but neglected some characteristics that are common to all species we examined. These are: anterior lobe of pronotum with lateral areas covered by silvery or whitish pubescence, which can vary in size (as in Figs 2A, 3A) (sometimes indistinct in older specimens, as in Figs 1A, 4A); middle tibia with a row of elongate dark-brown trichobothria-like setae on distal half, decreasing in size distally (Fig 2A); meso- and metasterna centrally with two pairs of small tubercles at intersegmental region (Fig 19C); abdominal sterna II–VI with short pubescence medially (Fig 11J); abdominal sterna II–VII each with two lateral, sub-ovate impressed furrows, the first located at intersegmental region (Fig 2C); and male proctiger without projections (Fig 10I).

**Discussion.** Prior to Andersen [2], *Angilia* was divided into three subgenera, *A*. (*Angilia*), *A*. (*Subangilia*) Poisson, 1955 and *A*. (*Adriennella*). The difference between *A*. (*Angilia*) and *A*. (*Subangilia*) was based on male abdominal segment VIII: species without lateral or ventral projections on this segment were classified in *A*. (*Angilia*), whereas those with projections were classified in *A*. (*Subangilia*). However, females were identical in the two subgenera, leading Andersen [2] to propose *A*. (*Subangilia*) as a junior synonym of *A*. (*Angilia*). He kept the genus divided into only two subgenera based on the antenna, pronotum, tibial grasping comb, and ratio between middle tibia and tarsus. However, after examining eleven species, we detected some inconsistencies in the two currently proposed subgenera. Our diagnoses of the two subgenera differ slightly from those of Andersen because the thickness of the antennomeres and the elevation of the pronotal disc can vary within a subgenus.

When present in species of the two currently valid subgenera, projections of male abdominal segment VIII are diagnostic, may be arranged into one or two pairs, be simple or bifid, and are usually located ventrolaterally. Such projections are not common in other Veliinae, being present only in some species of the American genus *Veloidea* Gould, 1934 and in *Paravelia confusa* (Hungerford, 1930) (H.D.D. Rodrigues, pers. obs.). Since the projections are located at different positions, it is likely that they evolved independently in the three genera.

After the taxonomic changes proposed in the present study, *Angilia* now comprises 23 species and two subspecies. Of these, 12 belong to the subgenus *A.* (*Angilia*), while 11 species and two subspecies are attributed to the subgenus *A.* (*Adriennella*). Geographically, 13 species and two subspecies occur in mainland Africa, three species in Madagascar, and seven species in Southeast Asia (Table 1).

**Table 1:** Checklist of species of *Angilia.*

Genus *Angilia* Stål, 1865	Distribution		
Subgenus *Angilia* (*Angilia*) Stål, 1865	Mainland Africa	Madagascar	Southeast Asia
*Angilia* (*Angilia*) *aeterna* Hoberlandt, 1946	X		
*Angilia* (*Angilia*) *albidotincta* (Stål, 1855)	X		
*= Angilia* (*Angilia*) *albidotincta ugandensis* Poisson, 1955 **n. syn**.	X		
*Angilia* (*Angilia*) *ambakakae* Poisson, 1952		X	
*Angilia* (*Angilia*) *collarti* Poisson, 1957	X		
= *Angilia* (*Angilia*) *collarti* Poisson, 1958a **n. syn.**	X		
*Angilia* (*Angilia*) *congoensis* Poisson, 1950	X		
*Angilia* (*Angilia*) *cruciata* Poisson, 1955	X		
*Angilia* (*Angilia*) *dubia* Poisson, 1942	X		
*Angilia* (*Angilia*) *kaokoveldi* Poisson, 1957	X		
= *Angilia kaokoveldi* Poisson, 1958a **n. syn.**	X		
= *Angilia* (*Angilia*) *albidotincta kaokoveldi* Poisson, 1958b **n. syn.**	X		
*Angilia* (*Angilia*) *kenyalis* Poisson, 1950	X		
*Angilia* (*Angilia*) *morogoro* Rodrigues, Sites & Moreira **n. sp.**	X		
*Angilia* (*Angilia*) *perplexa* Poisson, 1942	X		
*Angilia* (*Angilia*) *rhodesiensis* Poisson, 1955	X		
**Subgenus *Angilia* (*Adriennella*) Poisson, 1942**			
*Angilia* (*Adriennella*) *anderseni* Zettel & Hecher, 1998*			X
*Angilia* (*Adriennella*) *bertrandi* Poisson, 1963		X	
*Angilia* (*Adriennella*) *bispinosa* Andesern, 1981*			X
*Angilia* (*Adriennella*) *borneensis* Zettel & Hecher, 1998*			X
*Angilia* (*Adriennella*) *conradsi* Poisson, 1950	X		
*Angilia* (*Adriennella*) *igniventris* Rodrigues, Sites & Moreira **n. sp.**		X	
*Angilia* (*Adriennella*) *mazzoldii* Zettel & Hecher, 1998**			X
*Angilia* (*Adriennella*) *orientalis* Andersen, 1981**			X
*Angilia* (*Adriennella*) *philippiensis* Drake & Hoberlandt, 1953**			X
*Angilia* (*Adriennella*) *schoutedeni schoutedeni* Poisson, 1942	X		
*Angilia* (*Adriennella*) *schoutedeni camelus* Poisson, 1950	X		
*Angilia* (*Adriennella*) *trispinosa* Andersen, 1981*			X

*Angilia bispinosa group, **Angilia orientalis group, *sensu* Zettel & Hecher [3].

**Forewing maculation.** In his descriptions, Poisson used the shape and size of the white forewing maculae as a diagnostic feature. However, we observed significant intraspecific variation in such maculae. For example, the basal macula may vary in length; the mid-lateral macula may be present or absent in addition to varying in length; the medial macula may be present or absent and varies slightly in size; the apical maculae, consisting of a central macula surrounded by four other maculae posterolaterally, exhibit the greatest degree of variation: the posteromedial macula may be narrower, wider, or divided medially, and the posterolateral maculae often vary in size.

In contrast, in species such as *A. anderseni* and *A. bispinosa*, the two most distal posterolateral maculae are consistently fused with the posteromedial macula in all examined specimens. Thus, depending on the species, the maculation pattern may be stable and therefore diagnostic. Nonetheless, it is important to note that the maculation pattern changes according to the wing morph (macropterous vs. brachypterous). Intraspecific variation in forewing maculae has also been described in American Veliinae, including differences in size and even the absence of the apical macula (e.g., Rodrigues *et al.* [13]). Therefore, we consider that examining many specimens from different populations is necessary before using the maculae as diagnostic characters.

### Annotated list of taxa

#### *Angilia* (*Angilia*) Stål, 1865.

**Diagnosis.** This subgenus is characterized by the pronotum without a finger-like projection at the posterior margin (Fig 1A); the mesosternum without a pair of curved, longitudinal rows of rounded punctations; the metasternum without an irregular set of rounded punctations laterally; the female foretibial grasping comb distinctly shorter than that of the male, usually less than 1/3; and the middle tarsus subequal in length to the middle tibia.

#### *Angilia* (*Angilia*) *aeterna* Hoberlandt, 1946

(Figs 1, 24)

**Fig 5:**
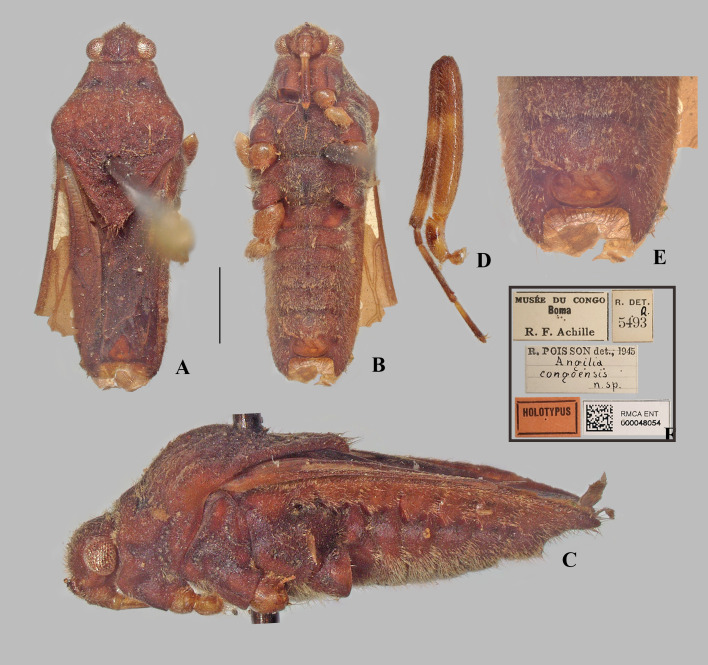
*Angilia* (*Angilia*) *congoensis* Poisson, 1950. (A) dorsal, (B) ventral and (C) lateral habitus of macropterous male lectotype (RMCA); (D) middle leg; (E) abdomen apex in ventral view; (F) lectotype labels. Scale bar = 1.0 mm and applies to Figs A–B.

**Fig 6:**
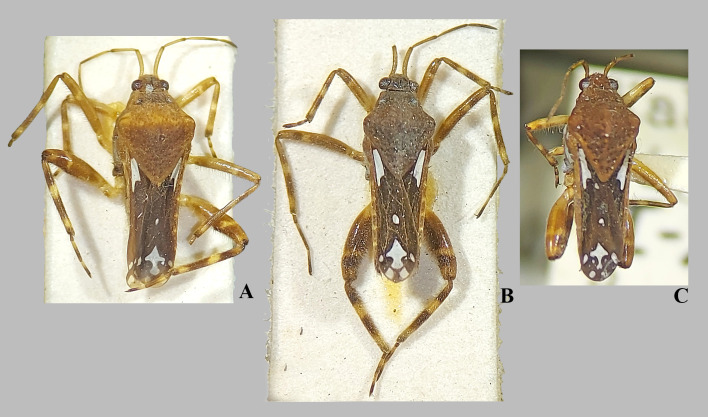
*Angilia* (*Angilia*) *congoensis* Poisson, 1950. (A–B) dorsal habitus of macropterous males from Democratic Republic of the Congo (NMNH); (C) dorsal habitus of macropterous female from Nigeria (NMNH).

**Fig 7:**
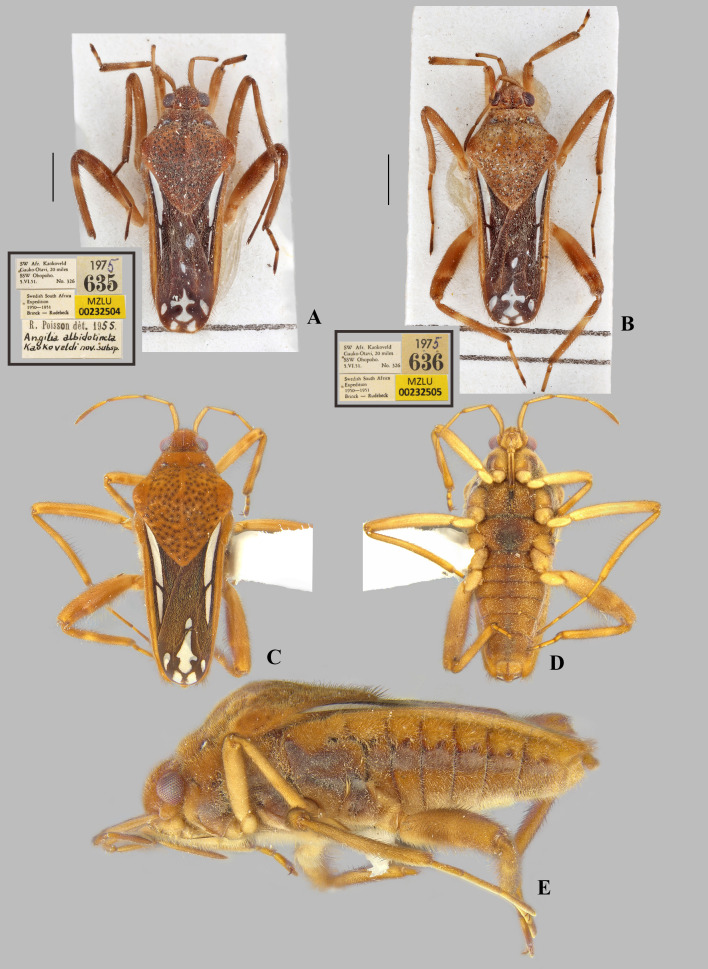
*Angili*a (*Angilia*) *kaokoveldi* Poisson, 1957. (A–B) dorsal habitus of macropterous female paratypes (MZLU); (C) dorsal, (D) ventral and (E) lateral habitus of macropterous female from Democratic Republic of the Congo (UMC). Figures A–B photographed by Rune Bygebjerg (© 2025 Biologiska museet, Lunds universitet). Made available by the Lunds universitet under Creative Commons Attribution 4.0 International Public License, CC-BY 4.0, https://creativecommons.org/licenses/by/4.0/legalcode. Scale bar = 1.0 mm and applies to Figs A–B.

**Fig 8:**
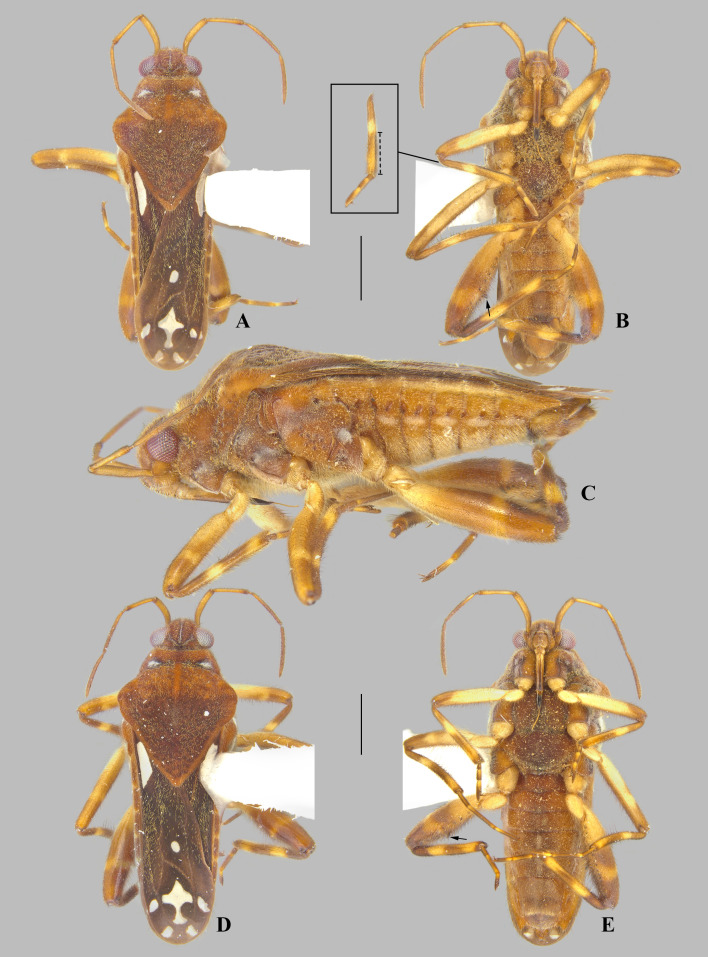
*Angilia* (*Angilia*) *morogoro* n. sp. (A) dorsal, (B) ventral and (C) lateral habitus of macropterous male holotype (UMC), inset shows part of foreleg, dashed line indicates length of tibial grasping comb, black arrow indicates large spine of hind femur; (D) dorsal and (E) ventral habitus of macropterous female paratype (UMC), black arrow indicates large spine of hind femur. Scale bars = 1.0 mm and apply to Figs A–B, D–E.

**Fig 9:**
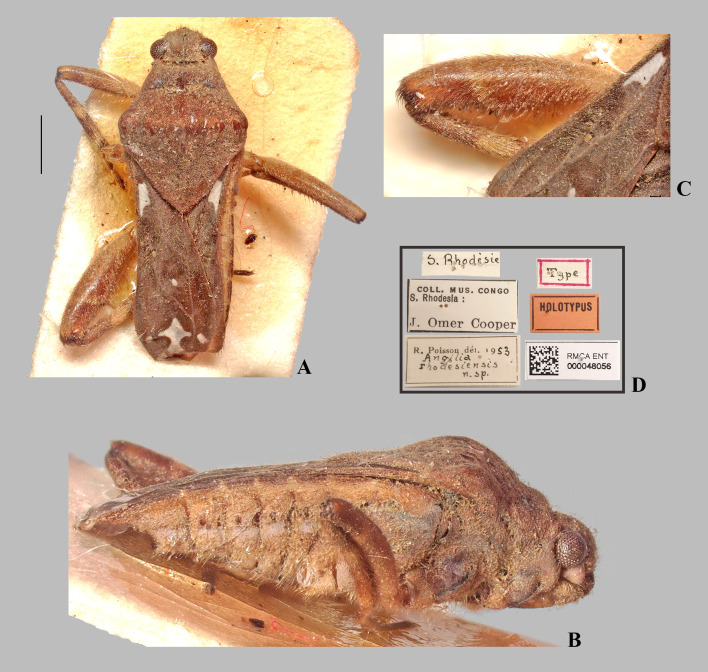
*Angilia* (*Angilia*) *rhodesiensis* Poisson, 1955. (A) dorsal and (B) ventral habitus of macropterous male holotype (RMCA); (C) hind femur in dorsal view; (D) holotype labels. Scale bar = 1.00 mm and applies to Fig A.

**Fig 10:**
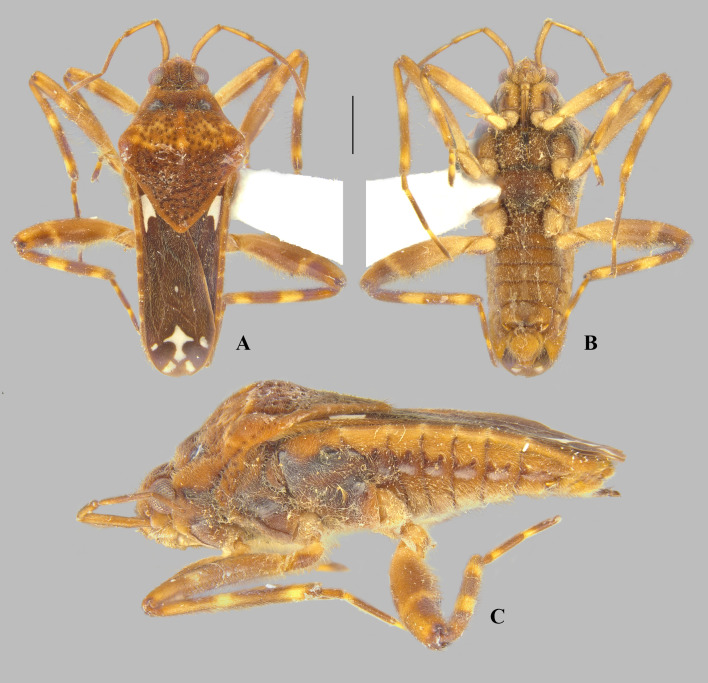
*Angilia* (*Angilia*) *rhodesiensis* Poisson, 1955. (A) dorsal, (B) ventral and (C) lateral habitus of macropterous male from Tanzania (UMC). Scale bar = 1.00 mm and applies to Figs A–B.

**Fig 11:**
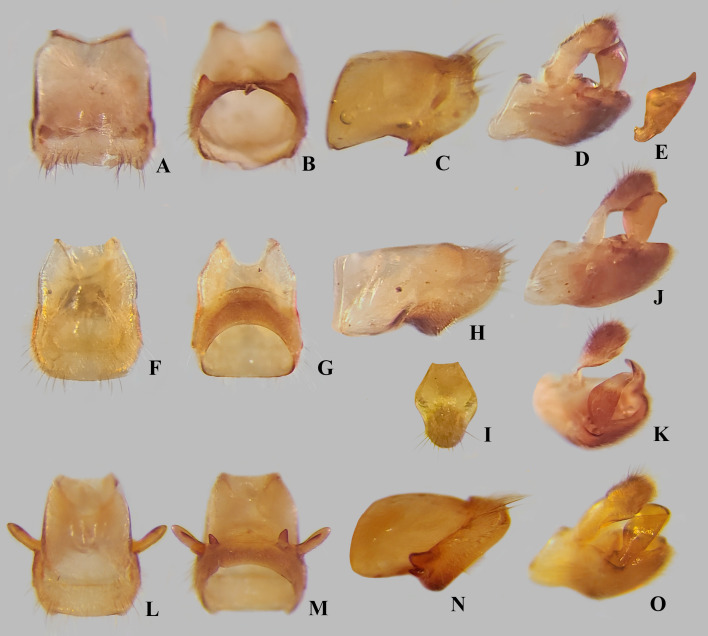
Male structures of *Angilia* spp. (A–E) *Angilia ambakakae* Poisson, 1952, (A) dorsal, (B) ventral and (C) lateral views of abdominal segment VIII; (D) genital capsule in lateral view; (E) left paramere in lateral view. (F–K) *Angilia morogoro* **n. sp.**, (F) dorsal, (G) ventral and (H) lateral views of abdominal segment VIII; (J) lateral and (K) posterolateral views of genital capsule; (I) proctiger in dorsal view. (L–O) *Angilia rhodesiensis* Poisson, 1955, (L) dorsal, (M) ventral and (N) lateral views of abdominal segment VIII; (O) genital capsule in lateral view.

**Fig 12:**
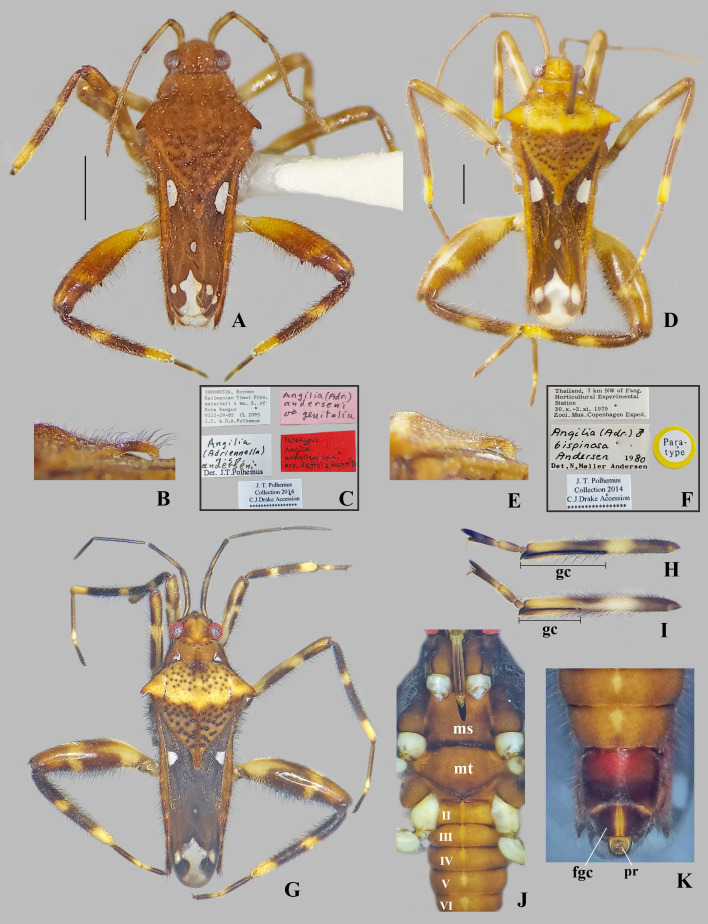
*Angilia* spp. (A–C) *Angilia* (*Adriennella*) *anderseni* Zettel & Hecher, 1998, (A) dorsal habitus of macropterous male paratype (NMNH) (genital capsule removed); (B) apex of pronotum in lateral view; (C) paratype labels. (D–K) *Angilia* (*Adriennella*) *bispinosa* Andersen, 1981, (D) dorsal habitus of macropterous male paratype (NMNH) (genital capsule removed); (E) apex of pronotun in lateral view; (F) paratype labels; (G) dorsal habitus of macropterous male from Vietnam (CEIOC); (H) male foretibia and tarsus; (I) female foretibia and tarsus; (J) ventral view of macropterous female from Vietnam; (K) terminal abdominal sterna of female. fgc = first gonocoxa, gc = grasping comb, ms = mesosternum, mt = metasternum, pr = proctiger. Scale bars = 1.00 mm and apply to Figs A, D.

**Fig 13:**
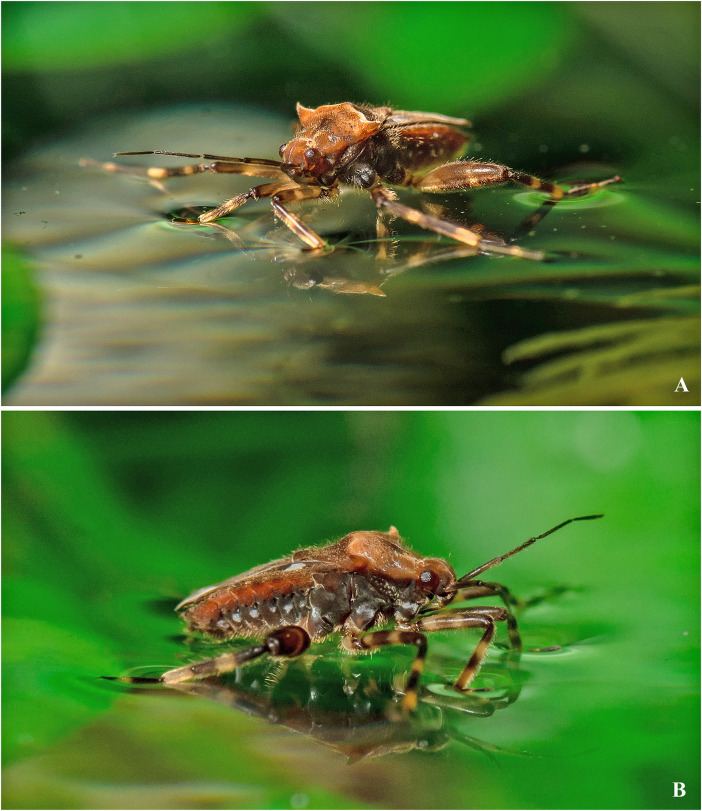
Living specimen of *Angilia* (*Adriennella*) *bispinosa* Andersen, 1981 from Vietnam. (A) anterolateral view, (B) lateral view. Photos courtesy of Arnaud Badiane (Institut de Génomique Fonctionnelle de Lyon, Lyon, France).

**Fig 14:**
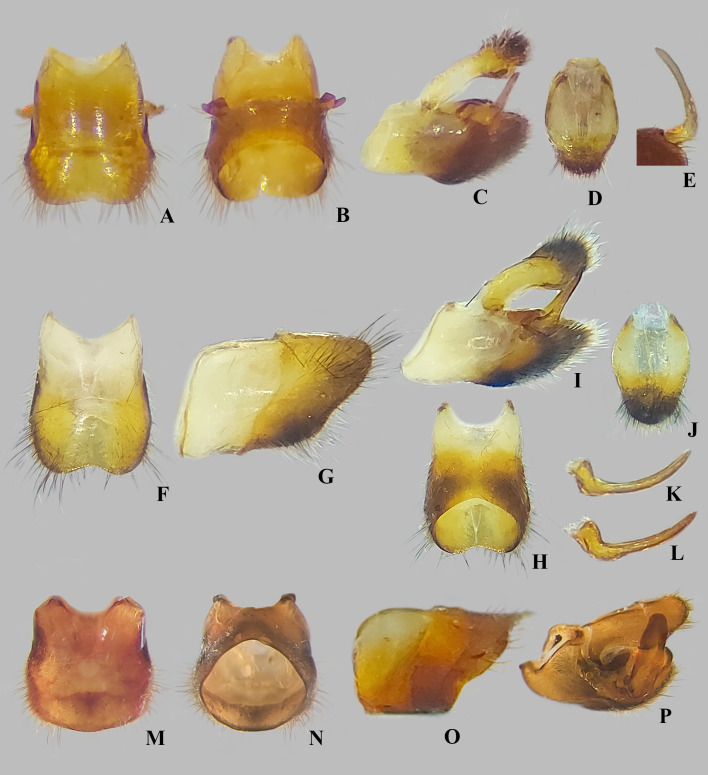
Male structures of *Angilia* spp. (A–E) *Angilia anderseni*, (A) dorsal and (B) ventral views of abdominal segment VIII; (C) genital capsule in lateral view; (D) proctiger in dorsal view; (E) paramere in posterodorsal view. (F–L) *Angilia bispinosa*, (F) dorsal, (G) lateral and (H) ventral views of abdominal segment VIII; (I) genital capsule in lateral view; (J) proctiger in dorsal view; (K–L) left paramere in dorsolateral views. (M–P) *Angilia orientalis*, (M) dorsal, (N) ventral and (O) lateral views of abdominal segment VIII; (P) genital capsule in laterodorsal view.

*Angilia* (*Angilia*) *aeterna* Hoberlandt, 1946: 55–58 (original description).

*Angilia* (*Angilia*) *aeternae*: Sallier Dupin (1979): 29 (incorrect subsequent spelling).

**Diagnosis.** This species ca be distinguished from other members of the subgenus by the elongate basal macula on the forewing, which originates from the humeral angle and extends beyond the posterior margin of the pronotum, the mid-lateral macula absent; the foretibial length 1.4 × the male grasping comb length; the hind femur ventrally with an irregular row of small dark-brown spines, including two large spines located slightly beyond the middle of the segment (Fig 1A); male abdominal segment VIII without projections; and the paramere apex acute.

**Discussion.** This species occurs just south of the Sahara Desert, in regions that partially correspond with the Sahel, with no records to date from areas further south on the African continent [14–18]. In the original description [16], the author does not mention a ventral spine on the middle femur, slightly beyond the middle of the segment (Fig 1A). This spine is not observed in other species of the genus and may therefore represent a diagnostic feature. However, the consistent presence of this structure must to be confirmed based on additional specimens, because such spines on the middle femur are uncommon in *Angilia* or other members of Veliinae (H.D.D. Rodrigues, pers. obs.). In the species key, *A. aeterna* shares the same pair of couplets as *A. albidotincta*, due to the absence of projections on the male abdominal segment VIII and the presence of one or two more robust spines ventrally on the hind femur. Both species can be distinguished by the maculae pattern on the forewing.

**Distribution.** Benin: Atakora, Zou [14]. Chad: Logone Oriental [15]. Niger [16,17]. Nigeria: Kaduna [15]. South Sudan [18] (Fig 23).

**Type material examined.** HOLOTYPE ♂ macropterous (NMPC), [NIGER], Maradi, Nigérie, Saha[r]a mer., X 1936, Škulina/ *Angilia aeterna* n. sp. ♂, L. Hoberlandt det./ Holotypus.

#### *Angilia* (*Angilia*) *albidotincta* (Stål, 1855)

(Figs 2, 3, 23A)

*Velia albidotincta* Stål, 1855: 46 (original description).

*Angilia* *albidotincta*: Stål 1865: 168 (combination change).

*Angilia* (*Angilia*) *albidotincta albidotincta*: Poisson 1955: 277 (subgeneric placement and subspecific status).

*Angilia* (*Angilia*) *albidotincta ugandensis* Poisson, 1955: 272–276 (original description) **n. syn.**

**Diagnosis.** This species can be distinguished from other members of the subgenus by the elongate basal macula on the forewing, which originates from the humeral angle, extends distinctly beyond the posterior margin of the pronotum, and is followed by another small macula (Figs 2A, 3A); the foretibial length 1.5 × the male grasping comb length; the hind femur ventrally with an irregular row of small dark-brown spines, including one or two large spines located slightly beyond the middle of the segment (Figs 2B, 3A, 3B); and male abdominal segment VIII without projections.

**Lectotype designation.** Stål [19] did not designate the holotype and did not indicate the number of specimens in the type series of this species, which we believe consists of three macropterous males deposited in the NHRS. One of these specimens received a “Typus” label (Fig 2) and the other two received “Paratypus” labels, but it is not clear when they were added and by whom. Because there is no subsequent designation of a type, not even implicitly by Poisson [7], we herein designate the “Typus” male as the lectotype of this species, while the “Paratypus” males are designated as paralectotypes.

**Discussion.** Poisson [7] described *A. albidotincta ugandensis* from two macropterous males and distinguished it from the nominal subspecies by three features: body size 5.5 (compared to 5.0 in *A. a. albidotincta*), the mid-lateral macula of the forewing more elongate, and a less pronounced hind femur spine. All of the differences used by Poisson [7] to separate these two subspecies are susceptible to variation, as observed within the genus and in other genera of the same subfamily. We examined a female from Tanzania very similar to the males of the type series of the nominal subspecies, but with small differences in the macula pattern on the forewing, such as the slightly longer mid-lateral macula (Fig 3A), one of the diagnostic features mentioned in the original description of *A. a. ugandensis*. We therefore consider these differences to be minor intraspecific variations and propose here *A. a. ugandensis* as a junior synonym of the nominal subspecies. Specimens reported by Poisson [4] actually belong to *A. cruciata* [7].

**Distribution.** Democratic Republic of the Congo: Haut-Katanga [20]. Ethiopia: Oromia [21,22]. South Africa: KwaZulu-Natal [23, present study], Limpopo [7, 19, present study]. Tanzania: Simiyu [present study]. Uganda: “Ouganda Central” [7]. Zambia: Central [24]. Zimbabwe [7] (Fig 23A).

**Type material examined.** LECTOTYPE [herein designated] of *Angilia albidotincta*, ♂ macropterous (NHRS), [SOUTH AFRICA], Caffraria [= Kaffraria] [**Limpopo**]/ J. Wahlb [= Johan August Wahlberg]/ albido-tincta Stål/ Typus/ 95 53/ NHRS-GULI 000110928. PARALECTOTYPES [herein designated]: same data as lectotype (2♂ macropterous NHRS).

**Additional material examined.** SOUTH AFRICA, **KwaZulu-Natal**: Africa, East Cape Prov., Umzimkulu [−30.26, 29.94], 13 March 1956, J. Omer-Cooper, No. 221/ *Angilia* (*Angilia*) sp., try *albidotincta* Stål/ *Angilia* (*Angilia*) *albidotincta* Stål, Det. J.T. Polhemus 1981/ J.T. Polhemus Collection 2014 C.J. Drake Accession (1♂ macropterous NMNH); same data, except: 14 March 1956, No. 223 (1♀ macropterous NMNH). TANZANIA, **Mara Region [Simiyu]**: Tairo, ca. 5 km east of Bunda, 24 July 2010, L-1146, R.W. Sites & A. Mbogho/ heavily vegetated pond margins, 02°33.827’S, 33°45.695’E, 1230 m (1♀ macropterous UMC).

#### *Angilia* (*Angilia*) *ambakakae* Poisson, 1952

(Figs 4, 11A–E, 23A)

*Angilia* (*Angilia*) *ambakakae* Poisson, 1952: 39–40 (original description).

**Diagnosis.** This species can be distinguished from congeners in the subgenus by the elongate basal macula on the forewing, which originates from the humeral angle and extends beyond the posterior margin of the pronotum, followed or not by another small macula (Figs 4A, D–E); the male hind trochanter bearing an irregular row of small, dark-brown pegs ventrally on the posterior margin (Fig 4B); the hind femur bearing an irregular row of small, dark-brown teeth or pegs ventrally, without large spines (Figs 4B, F); male abdominal segment VIII with a pair of small, acute, posteroventral projections (Figs 11B–C); and the paramere curved, elongate, widened at mid-length, tapered distally, with the apex acute (Figs 11D–E).

**Supplemental description. Macropterous male** (n = 7). Body length 4.60–4.88, body width 1.72–1.84. Foretibia length 2.2 × grasping comb length. Hind trochanter bearing irregular row of small, dark-brown pegs ventrally on distal half. Hind femur distinctly thicker than others, slightly dilated at posterior third, bearing irregular row of small, dark-brown teeth or pegs ventrally. Abdominal segment VIII slightly widened dorsally at posterior 1/4 (Fig 11A); pair of small, acute, posteroventral projections; posterior region with elongate golden setae dorsolaterally (Figs 11B–C). Proctiger without projections. Paramere curved, elongate, widened at mid-length, tapered distally, with apex acute (Figs 11D–E). **Macropterous female.** Foretibia length 4.8 × grasping comb length.

**Discussion.** This species is known only from Madagascar. Poisson [6] based his original description on two macropterous females, but did not indicate the depository of the type specimens. The females examined here match Poisson’s [6] original description, except for one specimen from the province of Menabe, which has a yellow region between the anterior and posterior lobes of the pronotum, a longer basal macula, and a mid-lateral macula on the forewing (Fig 4D). Thus, males were identified by association with females.

**Distribution.** Madagascar: Diana [present study], Melaky/ Menabe [6], Menabe, Sava [present study] (Fig 23A). Specimens from localities L-1853, 1854, 1855 (material examined) were collected on the banks of a rocky stream with vegetation, or in the flooded rice fields and overflow areas.

**Material examined.** MADAGASCAR, **Diana**: N. Madagascar, Marivorahona [−13.09, 49.11], nr. Ambilobe, a.h. 137, 5.xii.52, E.S. Brown/ *Angilia* (*Angilia*) *ambakakae* Poisson? det. I. Lansbury 1954/ *Angilia* (*Angilia*) *ambakakae* Poisson, Det. J.T. Polhemus/ J.T. Polhemus Collection 2014 C.J. Drake Accession (1♂, 1♀ macropterous NMNH). **Menabe**: Tulear Prov., river 10 km N. of Betsimba drill site, 105 km SE of Morondava 107 m, [−20.8239, 44.4144], XI-25–86, CL-2287, J.T. & D.A. Polhemus/ *Angilia ambakakae* Poisson Det. J.T. Polhemus/ J.T. Polhemus Collection 2014 C.J. Drake Accession (3♂, 1♀ macropterous NMNH); Tulear Prov., ponds 1 km N. of Betsimba drill site, 111 km SE of Morondava 183 m, [−20.8175, 44.4231], XI-25–86, CL-2288, J.T. & D.A. Polhemus/ *Angilia ambakakae* Poisson Det. J.T. Polhemus/ J.T. Polhemus Collection 2014 C.J. Drake Accession (5♂, 3♀ macropterous NMNH). **Antsiranana Prov. [Sava]**: 19 November 2014, colls: Sites, Gustafson & Miller, L-1854/ 42 m, 14°11.038’S, 50°1.500’E, flooded rice paddy & overflow (5♂, 4♀ macropterous UMC; 1♂, 1♀ macropterous CEIOC); Bemarivo River at Ambudipunt, L-1855, 10 November 2014/ 19 m, 14°12.292’S, 50°3.141’E, vegetated margins, coll: R.W. Sites (2♀ macropterous UMC); 14°3.552’S, 50°1.622’E, 10 Nov. 2014, R.W. Sites & K. Miller/ 65 m, unnamed rocky stream with vegetated margins L-1853, (1♂, 1♀ macropterous UMC).

#### *Angilia* (*Angilia*) *collarti* Poisson, 1957

*Angilia* (*Subangilia*) *collarti* Poisson, 1957: 175–177 (original description).

*Angilia* (*Subangilia*) *collarti* Poisson, 1958a: 54–55 (original description) (**new junior objective synonym and junior homonym**).

*Angilia* (*Angilia*) *collarti*: Andersen 1981: 343 (subgeneric change).

**Distribution.** Democratic Republic of the Congo: Lualaba, Tshopo [8,9] (Fig 24).

**Discussion.** This species was described twice by the same author, under the same name, in different publications [8,9]. Both type series are composed of the same specimens, a male and a female collected in the Democratic Republic of the Congo. However, in neither publication did Poisson designate the holotype, making these specimens syntypes; they are currently deposited at the RMCA. Thus, the description of Poisson [9] constitutes a junior objective synonym and a junior homonym of the original description of Poisson [8].

#### *Angilia* (*Angilia*) *congoensis* Poisson, 1950

(Figs 5–6, 22A)

*Angilia* (*Angilia*) *congoensis* Poisson, 1950: 82–83 (original description).

*Angilia* (*Subangilia*) *congoensis*: Poisson 1955: 278 (subgeneric change).

*Angilia* (*Angilia*) *congoensis*: Andersen 1981: 343 (subgeneric change).

**Diagnosis.** This species can be distinguished from congeners in the subgenus by the foretibia length 4 × the male grasping comb length; the basal white macula of the forewing almost reaching the base of the wing, with the posterior margin concave, exceeding the posterior margin of the pronotum (Figs 5A, 6); the hind trochanter with black denticles on the posterior margin – although not very evident, they can be observed in the lectotype (Fig 5B); the hind femur with small black spines along the posterior margin, except apically, without one or two larger spines on the posterior half; male abdominal segment VIII with two pairs of ventrolateral projections, with the ventral pair smaller and the lateral pair almost two times longer, both rounded apically (see Poisson [8]: 177, Fig 10b).

**Lectotype designation.** Poisson [5] described this species based on a male and female, both macropterous, collected in Boma, Democratic Republic of Congo. He indicated that these specimens were deposited in the “Musée du Congo”, now known as the RMCA. Poisson did not designate a holotype in the original description, nor in subsequent papers. However, the male is labeled “Holotypus” (Fig 5F) and the female “Paratypus”. The mere addition of these labels, which were probably not assigned by Poisson himself, does not formalize the type designation. Therefore, to ensure nomenclatural stability, the male is formally designated here as the lectotype and the female as the paralectotype.

**Discussion.** In the present study, only the male from Poisson’s [5] original series was examined (Fig 5). This specimen is in poor condition, lacking antennae, legs (except one middle leg), abdominal segment VIII, and genitalia (the last structure is probably at the NMNH, since this institution acquired Poisson’s slide collection). Thus, some diagnostic characters, such as in the hind leg and paramere, are based on the original description. This species is morphologically similar to *A. cruciata* Poisson, 1955 and *A. rhodesiensis* Poisson, 1955. They share the general coloration of the body, pattern of maculation on the forewing, and two pairs of projections on male abdominal segment VIII (as in Fig 11M). The paramere shape is more similar between *A. congoensis* and *A. cruciata*, and in both species the anterodorsal margin is distinctly notched and the apical third is distinctly tapered (see Poisson [7]: Figs 8a, 11a–b); however, these two conditions are more pronounced in *A. cruciata* compared to *A. congoensis*. In addition, the hind femur of *A. cruciata* has one or two larger spines on the posterior half in addition to the row of spines on the posterior margin (Poisson [4]: 156, fig 8a), whereas the larger spines are absent in *A. congoensis* (Poisson [5]: 82, Fig 16c).

*Angilia congoensis* and *A. rhodesiensis* can be distinguished by the following features: in *A. congoensis*, the ventral projection of male segment VIII is about half the size of the lateral projection, in addition to being slightly rounded apically (Poisson [8]: 177, Fig 10billustrated the mid-lateral); the hind femur has a row of small spines along the posterior margin, without one or two larger spines on the posterior half; and the basal macula on the forewing is more elongate and extends beyond the posterior margin of the pronotum (Fig 6). In *A. rhodesiensis*, the ventral projection of abdominal segment VIII is about three times smaller than the lateral projection, in addition to being acute apically (Figs 10L–M); the hind femur has, in addition to the row of spines on the posterior margin, one or two larger spines on the posterior half (Figs 9C, 10A–B); and the basal macula of the forewing ends at the same level as the posterior margin of the pronotum (Figs 9A, 10A).

**Distribution.** Chad: Logone Oriental [15]. Democratic Republic of the Congo: Haut-Katanga, Haut-Lomami, Kongo Central [5,25, present study]. Guinea: Nzérékoré [26]. Nigeria: Kaduna [15], Kogi [present study], Ogun [15] (Fig 23A).

**Type material examined.** LECTOTYPE [herein designated] ♂ macropterous (RMCA), [DEMOCRATIC REPUBLIC OF THE CONGO, **Kongo Central**], Musée du Congo, Boma [−5.84, 13.07], R.F. Achille/ R. det. R. 5493/ R. Poisson det. 1945 *Angilia congoensis* n. sp./ Holotypus/ RMCA ENT 000048054.

**Additional material examined.** DEMOCRATIC REPUBLIC OF THE CONGO, **Haut-Katanga**: Congo belge, P.N.U. [= Parc National d’Upemba] Kateke [rivière], s. affl. Lufira [= affluent de la Muovwe et sous-affluent droit de la Lufira] (960 m.) [−8.93, 26.70] 23-XI-5-XII-1948, Mis. G.F. de Witte, 1085a/ Coll. Mus. Tervuren/ R. Poisson det., 1951 *Angilia congoensis* Poiss./ JTP Coll. 84, Exch. from Tervuren/ J.T. Polhemus Collection 2014, C.J. Drake Accession (1♂ macropterous NMNH). **Haut-Lomami**: Congo belge, P.N.U. Mabwe r. E. lac Upemba [= rive est du lac Upemba] (585 m) [−8.67, 26.51] 11–26-I-1949, Mis. G.F. de Witte, 2212a/ Coll. Mus. Tervuren/ R. Poisson det., 1951 *Angilia congoensis* Poiss./ JTP Coll. 84, Exch. from Tervuren/ J.T. Polhemus Collection 2014, C.J. Drake Accession (1♂ macropterous NMNH). NIGERIA, **Kogi**: Kabba [7.82, 6.07], Nigeria, II-20–49, B. Malkin/ CJ Drake Coll. 1956/ *Angilia congoensis* Poisson, Drake (1♀ macropterous NMNH).

#### *Angilia (Angilia) cruciata* Poisson, 1955

*Angilia* *albidotincta*: Poisson 1942: 154–157 (misidentification).

*Angilia* *albidotincta*: Poisson 1954b: 7 (misidentification).

*Angilia* (*Angilia*) *cruciata* Poisson, 1955: 276 (original description).

**Distribution.** Ethiopia: “Ethiopie méridionale”. Ivory Coast. Uganda: “Ouganda Central” [4] (Fig 24).

**Discussion.** This species was identified twice by Poisson [4,25] as *A. albidotincta*. Later, after examining a male from the type series of *A. albidotincta*, Poisson [7] proposed the new species *A. cruciata* for his previously misidentified material. Poisson [4] provided several illustrations of *A. cruciata*, including ventral and lateral views of the abdomen, showing that male abdominal segment VIII has two pairs of ventrolateral projections, similar to those of *A. congoensis* and *A. rhodesiensis*. As Poisson [7] noted, at the time of the formal description of *A. cruciata*, the type specimens had already been lost in military events of 1944. Furthermore, the three males of the type series mentioned by Poisson [4] came from three countries (Ethiopia, Ivory Coast, and Uganda), and no specific localities were given. In particular, Ivory Coast in West Africa is geographically distant from the East African countries Uganda and Ethiopia. Therefore, the possibility that different species constituted the type series of *A. cruciata* cannot be excluded, and the inconsistency in the illustrations of the forewing by Poisson in [4] and [7] is evidence of this. Poisson ([4]: 154, Fig 6b) illustrated the mid-lateral macula, whereas Poisson ( [7]: 271, Fig 6c) did not.

***Angilia* (*Angilia*) *dubia* Poisson, 1942**

*Angilia dubia* Poisson, 1942: 157–159 (original description).

*Angilia* (*Subangilia*) *dubia*: Poisson 1955: 278 (subgeneric placement).

*Angilia* (*Angilia*) *dubia*: Andersen 1981: 343 (subgeneric change).

**Diagnosis.** According to the original description, this species can be distinguished from other members of the subgenus by the following features: foretibia 17 × the length of the grasping comb; forewing brachypterous, reaching half of abdominal tergum VI; hind femur bearing irregular rows of small, dark-brown spines ventrally, without a large spine; and body length 5.25 [4].

**Distribution.** Democratic Republic of the Congo: Haut-Katanga [4] (Fig 24).

**Discussion.** The description of this species was based on a single brachypterous female from Democratic Republic of the Congo. Poisson [4] noted that it may simply be a brachypterous female of *A. albidotincta*. However, according to the illustrations in the original description, the length of the grasping comb of the foretibia and the spines arrangement of the hind femur do not resemble specimens of *A. albidotincta*. This species was tentatively placed in the subgenus *Subangilia* although it is known from only one female and this subgenus was characterized by structures of male abdominal segment VIII [7]. When Andersen [2] considered *Subangilia* invalid, it was placed back in the nominal subgenus.

#### *Angilia* (*Angilia*) *kaokoveldi* Poisson, 1957

(Figs 7, 24)

*Angilia* (*Angilia*) *kaokoveldi* Poisson, 1957: 172–174 (original description).

*Angilia kaokoveldi* Poisson, 1958a: 53–54 (original description) (**new junior objective synonym and junior homonym**).

*Angilia* (*Angilia*) *albidotincta kaokoveldi* Poisson, 1958b: 172–174 (original description) (**new junior subjective synonym and junior homonym**).

**Diagnosis.** This species can be distinguished from other members of the subgenus by the elongate basal macula on the forewing, which originates from the humeral angle, extends distinctly beyond the posterior margin of the pronotum, and is followed by another narrow, elongate macula (Figs 7A–C); the hind femur ventrally with an irregular row of small dark-brown spines, without large spines (Figs 7A–D); and male abdominal segment VIII without projections.

**Discussion.** This species was described three times by the same author, under the same name, in different publications [8–10]. In one of these publications, Poisson [10] treated it as a subspecies, *Angilia albidotincta kaokoveldi*, based on two females from Kaokoveld, now part of Namibia. However, although it was printed in 1957, the publication was delayed and it was officially released on February 28, 1958 (see the website link: http://hbs.bpbmwebdata.org/dating/sherbornia/safranimlife.html, accessed May 5, 2025). At the same time, after examining male specimens, Poisson described this taxon as a species in two separate publications [8,9]: *Angilia kaokoveldi*. The type series of these two publications includes, in addition to the two females from Namibia, males collected in southern Angola.

Of these three publications, the first to be officially published was Poisson [8], and it was the only one in which he designated a male holotype. In the other two publications, no holotype was formally designated, so all the specimens cited are syntypes. We had the opportunity to examine two female paratypes of Poisson [8] deposited at the MZLU. Although Poisson indicated the RMCA as the repository of the holotype, this specimen is not deposited there; in that collection there are two macropterous specimens with the following labels: “Coll. Mus. Congo, S. Angola: Namakunde Front. Ovampoland V–VI 1948, C. Koch/ *Angilia kaokoveldi* Poiss./ R. Det. 6987 E/ Paratypus”. However, we were not able to examine these specimens, which probably belong to the original series of Poisson [8,9].

Since the type series of Poisson [9] includes the same specimen desiginated as the holotype in Poisson [8], the species described in [9] represents a junior objective synonym, in addition to a junior homonym, since it has the same name. Further, the two syntype females examined in Poisson [10] were also included in the type series of Poisson [8] as paratypes (one of them as ‘allotype’). However, since these females do not correspond to the same specimen as the holotype male, this description constitutes a junior subjective synonym in addition to a junior homonym.

This species has morphological similarities with *A. albidotincta*, including the presence of an elongated basal macula followed by a mid-lateral macula on the hemelytra, the absence of projections on male abdominal segment VIII, and the shape of the paramere. However, in *A. kaokoveldi*, the mid-lateral macula on the hemelytra is more elongate, and the hind femur of both males and females has an irregular row of small, dark-brown spines along the ventral margin (Fig 6). In *A. albidotincta*, the mid-lateral macula is smaller, and the hind femur has a row of more robust spines in addition to one or two more developed spines; the femur is also slightly thicker (Figs 2A–C, 3A–B). A female from the Democratic Republic of the Congo has been provisionally identified as *A. kaokoveldi*, based on differences in the posterior maculae on the hemelytra and the thickness of the hind femur. More specimens are needed for a better assessment of intraspecific variation. However, the relative geographic proximity of the localities and the similarity in the general morphological pattern suggest conspecificity with *A. kaokoveldi*.

**Distribution.** Angola: Cunene [8,9]. Democratic Republic of the Kongo: Kongo Central [present study]. Namibia: Kunene [8–10, present study], Ohangwena [present study] (Fig 24).

**Type material examined.** PARATYPES: [NAMIBIA, **Kunene**] SW Afr., Kaokoveld, Gauko-Otavi, 20 miles SSW Ohopoho [−18.30, 13.65], 5.VI.51, No. 326/ Swedish South Africa Expedition, 1950–1951, Brinck–Rudebeck/ R. Poisson det. 1955 *Angilia albidotincta kaokoveldi* nov. subsp./ 1975, 635/ MZLU 00232504 (2♀ macropterous MZLU).

**Additional material examined.** DEMOCRATIC REPUBLIC OF THE CONGO, **Kongo Central**: Bas Congo Province, Fwadiagombe River, ca. 4 km SW of Snell-Kwilu power station, 7 August 2012, L-1444/ Sites, Shepard & Pwema, 05°37.689’S, 14°14.368’E, 329 m, vegetated margins of gravel stream (1♀ macropterous UMC). NAMIBIA, **Ohangwena**: S.W. Africa, Oshikango [−17.40, 15.89] 3200 ft., 9-III-1970, E.S. Ross/ JTP Coll., Exch. from CAS/ JT Polhemus Collection 2014, C.J. Drake Accession (1♀ macropterous NMNH).

#### *Angilia (Angilia) kenyalis* Poisson, 1950

*Angilia* (*Angilia*) *kenyalis* Poisson, 1950: 81–82 (original description).

*Angilia* (*Subangilia*) *kenyalis*: Poisson 1955: 278 (subgeneric change).

*Angilia* (*Angilia*) *kenyalis*: Andersen 1981: 343 (subgeneric change).

**Distribution.** Kenya [5] (Fig 24).

***Angilia* (*Angilia*) *morogoro* Rodrigues, Sites & Moreira n. sp.**

(Figs 8, 11F–K, 23A)

urn:lsid:zoobank.org:act:F82B4304-2459–4291-8C3F-87A0CD98DE8E

**Description.** Macropterous male: HOLOTYPE (Figs 8A–C), length 4.96; maximum width 1.76. PARATYPES (n = 3), length 4.72–5.36 (mean = 4.97), maximum width 1.76–1.84 (mean = 1.81).

*Color* General dorsal coloration brown, with yellow-banded legs; ventrally dark-brown on thoracic sterna and brown to light-brown on abdominal sterna. Antenna yellowish-brown. Labium with articles I–II and basal part of III yellowish-brown, most of III brown, IV black. Forewing brown to dark-brown, veins pale-brown; an elongate white macula at base, starting away from humeral angle and slightly exceeding posterior margin of pronotum, tapered posteriorly; a small, rounded, white macula at mid-length of posterior discal cell; and one large, irregularly shaped macula and four smaller, rounded maculae at apex of wing (Fig 8A). Legs with coxae, trochanters, basal 2/3 of fore- and middle femora, and basal 1/3 of hind femur yellowish; apical third of fore- and middle femora, apical half of hind femur brown, and tibiae brown; each femur with a faint band at distal half; each tibia with two yellow bands, one at basal third and another at apex; tarsomeres brown with apical parts of articles yellow.

*Pilosity*. Head, antenna, pronotum, legs, lateral edge of abdominal laterotergites, pleura, and abdominal sterna covered by fine golden pubescence intermixed with elongate dark-brown setae. Anterior lobe of pronotum with small, subtriangular, frosty pubescence posterolaterally. Forewing with elongate golden setae on basal two thirds. Thoracic pleura and acetabula with faint, irregular-shaped, frosty pubescence. Abdominal sterna II–VII below impressed furrows with longitudinal row of frosty pubescence.

*Head* Short, strongly deflected in front of eyes, with impressed median line; pair of narrow indentations near mesal margins of eyes; length 0.50, width including eyes 0.98; eye width 0.28. Ocular setae present. Antenniferous tubercle wide, swollen. Antennomere I thicker than others, curved laterally; II slightly thicker than III and IV; III and IV subequal in width; IV filiform; length of antennomeres I–IV, 0.68, 0.60, 0.80, 0.82. Labium reaching middle of mesosternum.

*Thorax* Pronotum length along midline 2.00, maximum width 1.76; humeri raised; posterior lobe covered by rounded punctations, with a low carina along midline, lacking distinct elevation between humeral angles, distal tip narrowly rounded, without finger-like projection medially. Forewing reaching tip of abdomen, covering genital capsule, with four closed cells. Proepimeron with an irregular row of rounded punctations. Meso- and metepimeron each with scattered rounded punctations. Meso- and metasterna centrally without two pair of small tubercles on intersegmental region. Metasternum with posterior margin convex.

*Legs*. Foretibia with grasping comb (0.68 long) occupying 2/3 of its length (Fig 8B). Middle tibia slightly curved, with a row of elongate dark-brown trichobothria-like setae on distal half, decreasing in size distally. Hind trochanter bearing small, dark-brown teeth or pegs ventrally. Hind femur distinctly thicker than others, slightly dilated at mid-length, bearing irregular rows of small, dark-brown spines ventrally, including one large spine located slightly beyond middle of segment (Fig 8B). Hind tibia with scattered, dark-brown pegs ventrally. Leg measurements as follows: foreleg, femur 1.34, tibia 1.26, tarsomeres 1–3, 0.08, 0.18, 0.34; middle leg, femur 1.82, tibia 1.70, tarsomeres 1–3, 0.30, 0.66, 0.57; hind leg, femur 1.84, tibia 1.62, tarsomeres 1–3, 0.18, 0.40, 0.40.

*Abdomen* Lateral margins almost parallel. Laterotergites not elevated, without black denticles. Laterally, on region of insertion of lateral abdominal muscles, with a pair of sub-ovate furrows on sterna II–VII. Laterotergite VII with posterior corner tapering to apex, not spinose. Sterna IV–V with longitudinal areas with few setae at midline. Sternum VII with posteromedial margin truncate, slightly extended distally (Fig 8B). Segment VIII poorly widened on posterior half dorsally, without pairs of projections ventrolaterally (Figs 11F–H). Proctiger with golden setae posteriorly (Fig 11I). Paramere elongate, curved, widened at mid-length, tapering to a truncated apex (Figs 11J–K).

**Macropterous female.** Paratype (n = 1), length 5.28; maximum width 2.00. Similar to macropterous male in general structure and coloration (Figs 8D–E), except as follows: female grasping comb distinctly shorter, foretibia length 6.8 × grasping comb; hind trochanter without dark-brown denticles; laterotergite VII with posterior corner narrowly rounded, not spinose; abdominal sternum VII with posteromedial margin slightly concave; first gonocoxae plate-like; proctiger globose, posteriorly oriented.

**Distribution and habitat.** This species is known from three localities in Morogoro region, mid-eastern Tanzania (Fig 23A). The known habitats of this species consist of vegetated margins of rocky, sandy or gravel bed streams.

**Diagnosis.** This species can be distinguished from congeners in the subgenus by the basal macula of the forewing not reaching the base of the wing and slightly exceeding the posterior margin of pronotum (Figs 8A, D); the hind femur, in both sexes, bearing irregular rows of small, dark-brown spines ventrally, including one large spine located slightly beyond the middle of the segment (Figs 8B, E); male abdominal sternum VII with the posteromedial margin truncate, slightly extended distally (Fig 8B); male abdominal segment VIII without ventrolateral projections (Figs 11F–H); and the paramere widened at the mid-length, tapering to the truncated apex (Figs 11J–K).

**Discussion.** This species has no projections or processes on male abdominal segment VIII. In the subgenus *A*. (*Angilia*), this structure is also absent in the following species: *A. aeterna* Hoberlandt, 1946, *A. albidotincta*, and *A. kaokoveldi* Poisson, 1957. However, the parameres of the first two are distinctly tapered towards the apex, arrow-shaped, as illustrated by Hoberlandt ( [15]: 57, Figs 2d–e) and Poisson ([7]: 273, Figs 7e–f), respectively. The paramere of *A. kaokoveldi* is somewhat similar to that of *A. morogoro* **n. sp.**, although a difference in general shape is evident, mainly at the apex; also, in *A. kaokoveldi* the mid-lateral macula is present on the forewing (absent in *A. morogoro* **n. sp.**) and the hind femur does not have a spine larger than the others (present in *A. morogoro* **n. sp.**). There are two other species of *A*. (*Angilia*) from mainland Africa known only from females: *A. dubia* Poisson, 1942 and *A. perplexa* Poisson, 1942. They differ from *A. morogoro* **n. sp.** in the absence of the large spine on the hind femur. In addition, *A. perplexa* has a distinctly elevated pronotal disc between the humeral angles (see Poisson [4]: 159, Fig 13b), a feature not present in *A. morogoro* **n. sp.**

**Etymology.** The specific epithet refers to the region where the type series was collected.

**Type material examined.** HOLOTYPE, ♂ macropterous (UMC), TANZANIA, **Morogoro** Region, Kisawasawa River at Kisawasawa, 8 August 2013, L-1625, Sites, Mbogho, Rwegasira/ 326 m, 07°53.596’S, 36°52.311’E, vegetated margins of sandy stream. PARATYPES: same data as holotype (1♂ macropterous UMC); Morogoro Region, Kilombero District, Mwaya River at Mangula, 7 August 2013, L-1623, Sites, Mbogho, Rwegasira/ 313 m, 07°50.945’S, 36°53.201’E, muddy, vegetated margin of sand, gravel stream (1♀ macropterous UMC); Morogoro Region, Sonjo River at Sonjo, 8 August 2013, colls: Sites, Mbogho & Rwegasira, L-1626/ 320 m, 07°48.490’S, 36°53.785’E, vegetated margins of rocky stream (2♂ macropterous UMC).

#### *Angilia (Angilia) perplexa* Poisson, 1942

*Angilia perplexa* Poisson, 1942: 159–161 (original description).

*Angilia* (*Adriennella*) *perplexa*: Poisson 1955: 278 (subgeneric placement).

*Angilia* (*Angilia*) *perplexa* (**subgeneric change**).

**Diagnosis.** According to the original description, this species can be distinguished from other members of the subgenus by the following features: foretibia 7 × the length of the grasping comb; forewing basal macula small, not extending to the humeral angle, without mid-lateral macula; hind femur bearing irregular rows of small, dark-brown spines ventrally, without a large spine; and body length 5.50 [4].

**Distribution.** Democratic Republic of the Congo: Kasaï [4] (Fig 24).

**Discussion.** Poisson [4] described this species based on a single female from the Democratic Republic of the Congo, the only specimen known to date. Later, based mainly on the elevation of the pronotal disc between the humeral angles, Poisson [7] placed this species in the subgenus *Adriennella*. However, in the drawing of the pronotum in dorsal view included in the original description (see Poisson [4]: 159, fig 13a), the posterior margin does not have a finger-like process. Furthermore, the grasping comb is short, with the length of the foretibia 7 × the length of the grasping comb (see Poisson [4]: 161, fig 15b). Thus, this species is not a member of *Adriennella* and is herein transferred to the subgenus *Angilia*.

#### *Angilia* (*Angilia*) *rhodesiensis* Poisson, 1955

(Figs 9–10, 11L–O, 23A)

*Angilia* (*Subangilia*) *rhodesiensis* Poisson, 1955: 270–272 (original description).

*Angilia* (*Angilia*) *rhodesiensis*: Andersen 1981: 343 (subgeneric change). 

**Diagnosis.** This species can be distinguished from congeners in the subgenus by the foretibia length 2.4 × the male grasping comb length; the basal macula of the forewing not reaching the base of the wing, with the posterior margin concave and at the same level as the posterior margin of the pronotum (Figs 9A, 10A); the hind trochanter with a set of black denticles on the posterior margin; the hind femur with an irregular row of small black spines along the posterior margin, with one or two larger spines on the posterior half (Figs 9C, 10A–B); male abdominal segment VIII with two pairs of ventrolateral projections, with the ventral pair smaller and acute apically, while the lateral pair is almost three times longer and rounded apically (Figs 11L–M). The paramere is widened at mid-length, tapering to an acute apex (Fig 11O).

**Discussion.** Poisson [7] described this species based on a male and a female, both macropterous, collected in the former Southern Rhodesia, now Zimbabwe. Poisson did not provide further information regarding the type locality. The type series is deposited at the RMCA and we had the opportunity to examine the male holotype (Fig 9). The specimen is in poor condition, with the abdomen glued to cardboard, without antennae and with only one fore, one middle and one hind leg. Abdominal segment VIII and the genital capsule have been dissected but are not attached to the specimen (these structures are probably in Poisson’s slide collection purchased by the NMNH). Despite the difficulties in observing the hind femur, this species clearly has a series of small spines along the posterior margin, including a larger spine on the distal half (Fig 9C). This species is morphologically similar to *A. congoensis* and *A. cruciata*. For a comparison, see discussion under *A. congoensis*.

**Distribution and habitat.** Tanzania: Rukwa [present study]. Zimbabwe [7, present study] (Fig 23A). The Tanzanian specimens were collected in a clear stream with marginal vegetation and rocks.

**Type material examined.** HOLOTYPE, ♂ macropterous (RMCA), [ZIMBABWE], S. Rhodésie/ Coll. Mus. Congo, S. Rhodesia: J. Omer Cooper/ R. Poisson dét. 1953, *Angilia rhodesiensis* n. sp./ Type/ Holotypus/ RMCA ENT 000048056.

**Additional** **material examined.** TANZANIA, **Rukwa Region**: Kamawe River at Puwi, 3August 2010, L-1205, R.W. Sites & A. Mbogho/ clear stream, marginal vegetation, some rocks, 08°21.029’S, 31°50.054’E, 1598 m. (2♂ macropterous UMC).

#### *Angilia* (*Adriennella*) Poisson, 1942

**Diagnosis** This subgenus is characterized by the pronotum with a finger-like projection on the posterior margin (as in Figs 12A, D, G); the grasping comb of the female foretibia almost as long as that of the male (Figs 12H–I); and the middle tarsus about 2/3 the length of the middle tibia.

**Discussion**. Initially, when examining some species of this subgenus, we thought that the mesosternum with a pair of curved, longitudinal rows of rounded punctations, and the metasternum with an irregular set of rounded punctations laterally were diagnostic for *A.* (*Adriennella*) (Fig 20C). However, some species from Southeast Asia lack these punctations, such as *A. anderseni* and *A. bispinosa* (Fig 12J). It is necessary to study these features in other species not examined here to determine if they can be used to distinguish groups of species within the subgenus.

#### *Angilia* (*Adriennella*) *anderseni* Zettel & Hecher, 1998

(Figs 12A–C, 14A–E, 25A)

**Fig 15:**
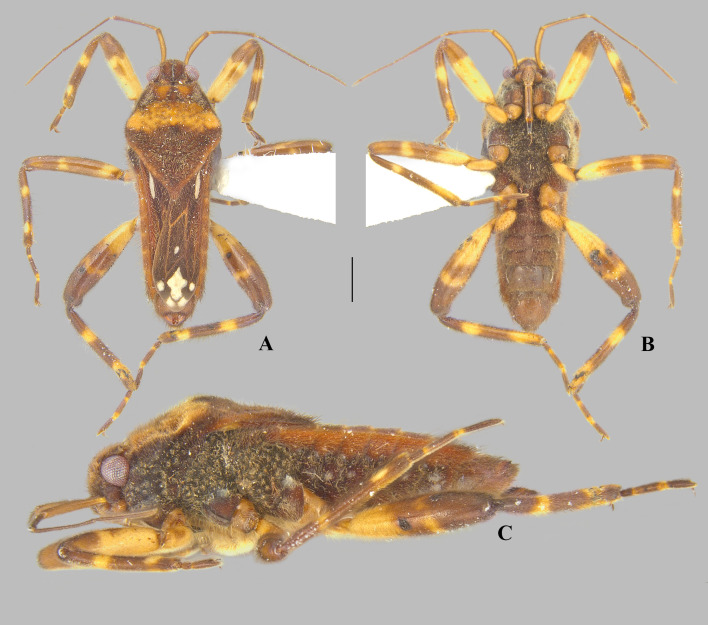
*Angilia* (*Adriennella*) *bertrandi* Poisson, 1963. (A) dorsal, (B) ventral and (C) lateral habitus of macropterous male from Haute Matsiatra (UMC). Scale bar = 1.00 mm and applies to Figs A–B.

**Fig 16:**
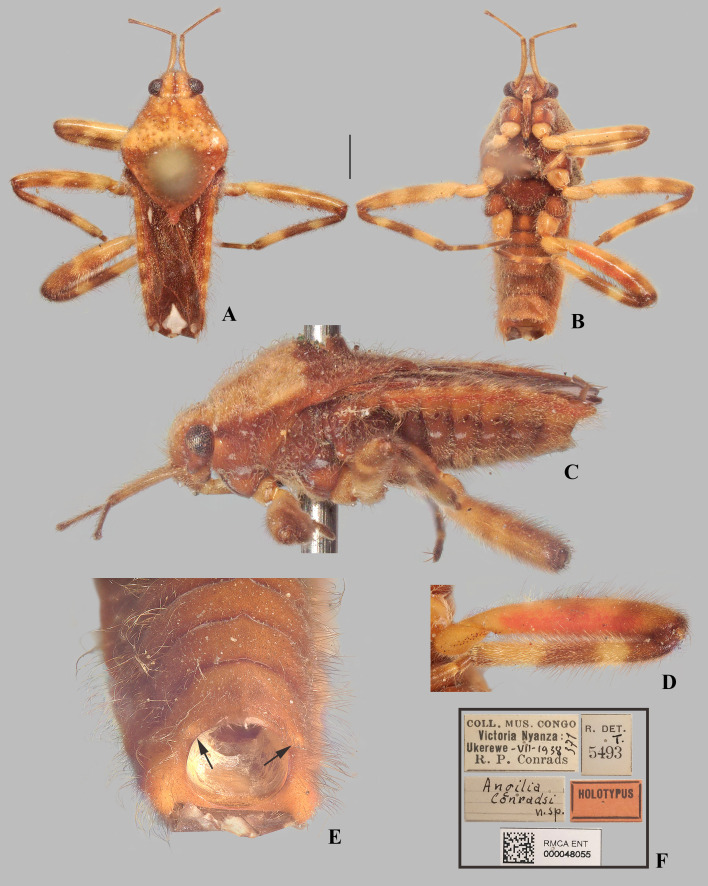
*Angilia* (*Adriennella*) *conradsi* Poisson, 1950. (A) dorsal, (B) ventral and (C) lateral habitus of macropterous male lectotype (RMCA); (D) hind leg in ventral view; (F) lectotype labels. Scale bar = 1.00 mm and applies to Figs A–B.

**Fig 17:**
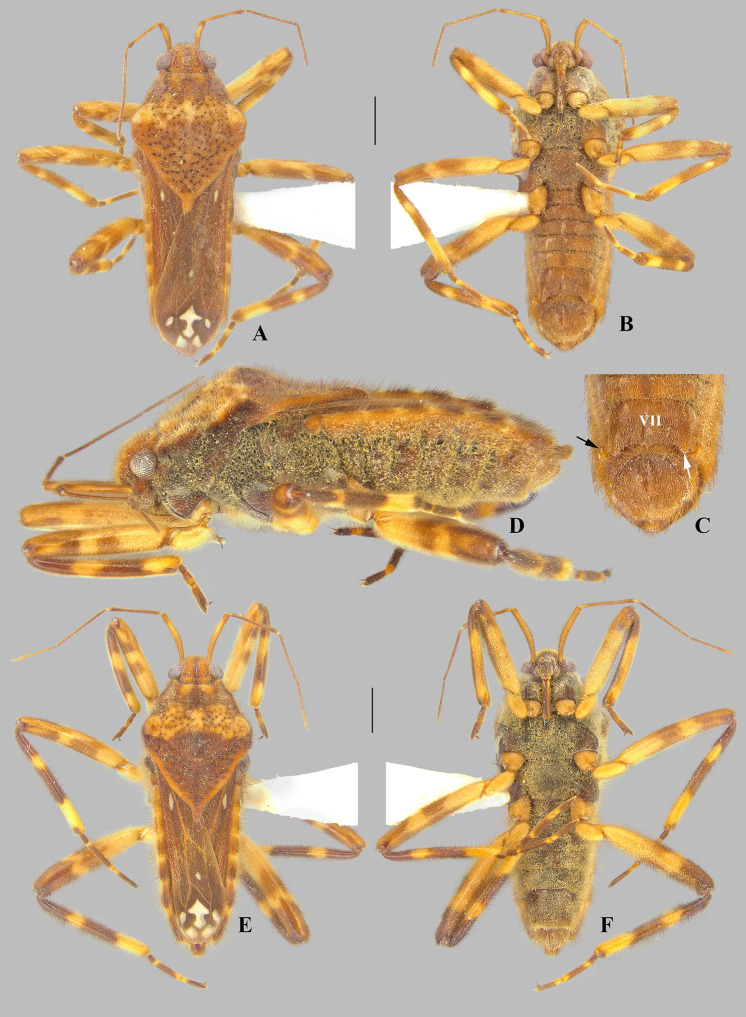
*Angilia* (*Adriennella*) *conradsi* Poisson, 1950. (A) dorsal and (B) ventral habitus of macropterous male from Tanzania (UMC); (C) terminal abdominal sterna of male, white arrow indicates posterolateral corner of segment VII, black arrow indicates lateral projection of segment VIII; (D) lateral, (E) dorsal and (F) ventral habitus of macropterous female from Congo (UMC). Scale bars = 1.00 mm and apply to Figs A–B, E–F.

**Fig 18:**
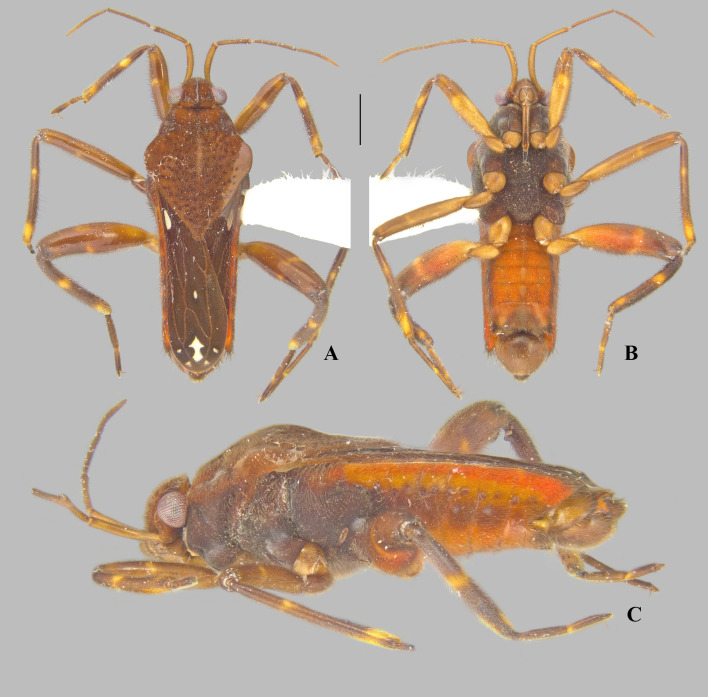
*Angilia* (*Adriennella*) *igniventris* n. sp. (A) dorsal, (B) ventral and (C) lateral habitus of macropterous male holotype (UMC). Scale bar = 1.00 mm and applies to Figs A–B.

**Fig 19:**
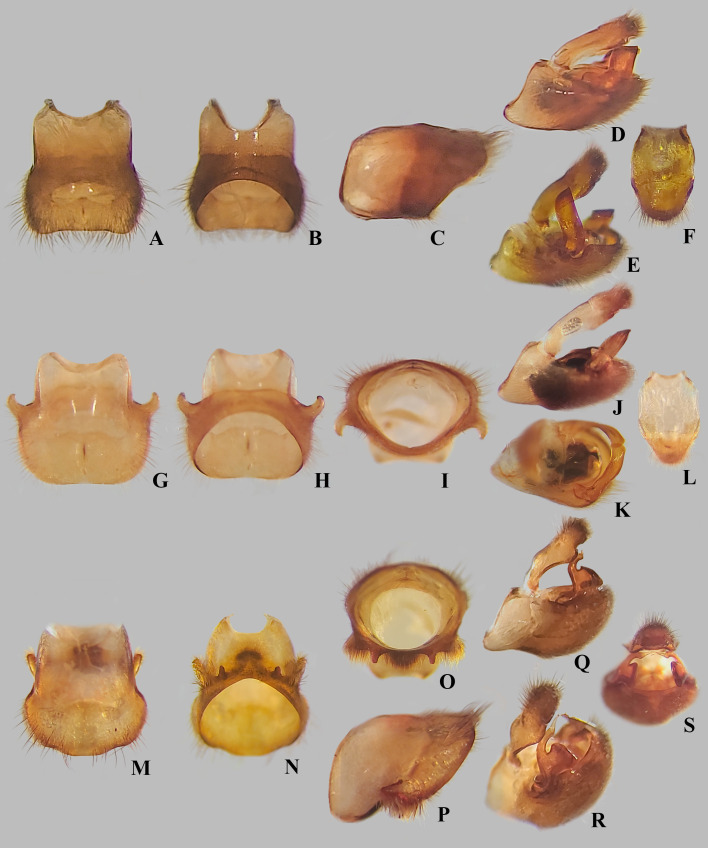
Male structures of *Angilia* spp. (A–F) *Angilia bertrandi*, (A) dorsal, (B) ventral and (C) lateral views of abdominal segment VIII; (D) genital capsule in lateral view; (E) genital capsule in lateral view with paramere raised; (F) proctiger in dorsal view. (G–L) *Angilia conradsi*, (G) dorsal, (H) ventral and (I) posterior views of abdominal segment VIII; (J) genital capsule in lateral view; (K) genital capsule in dorsolateral view; (L) proctiger in dorsal view. (M–S) *Angilia igniventris* **n. sp.**, (M) dorsal, (N) ventral, (O) posterior and (P) lateral views of abdominal segment VIII; (Q) lateral, (R) posterolateral and (S) posterior views of genital capsule.

**Fig 20:**
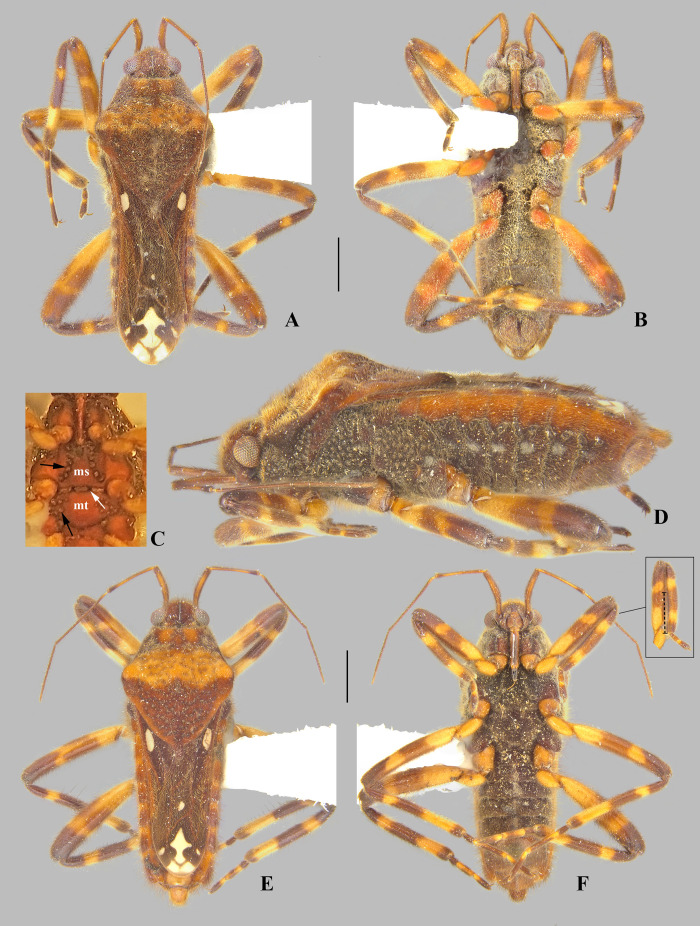
*Angilia* (*Adriennella*) *orientalis* Andersen, 1981. (A) dorsal and (B) ventral habitus of macropterous male from Thailand (UMC); (C) ventral view of thorax, black arrows indicate rounded punctations, white arrow indicates small tubercle on intersegmental region of meso- and metasterna; (D) lateral, (E) dorsal and (F) ventral views of macropterous female from Thailand (UMC), inset shows part of foreleg, dashed line indicates length of tibial grasping comb. ms = mesosternum, mt = metasternum. Scale bars = 1.00 mm and applies to Figs A–B, E–F.

**Fig 21:**
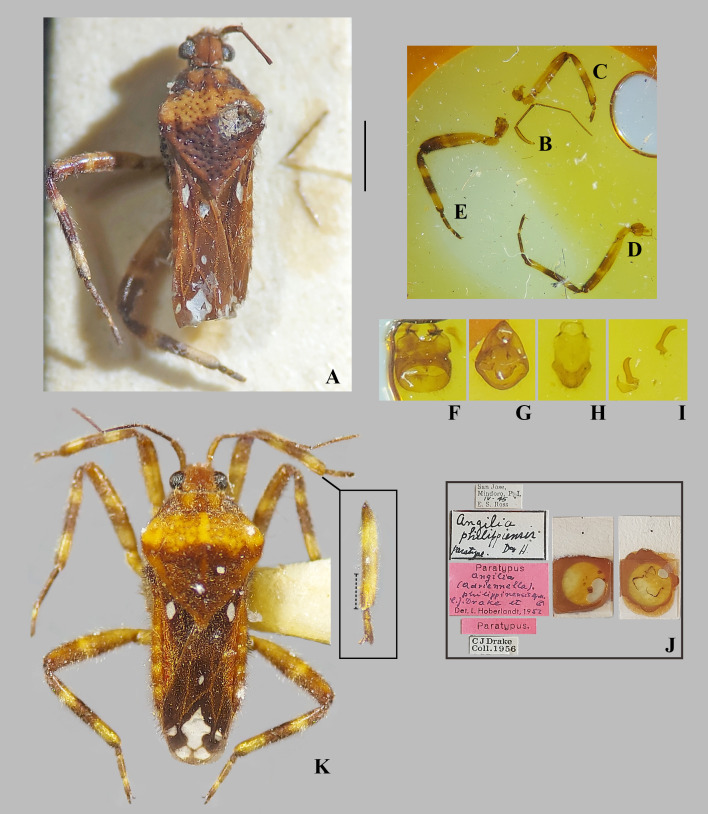
*Angilia* (*Adriennella*) *philippiensis* Drake & Hoberlandt, 1953. (A–J) macropterous male paratype (NMNH); (A) dorsal habitus; (B) left antenna; (C) foreleg in dorsal view; (D) middle leg in dorsal view; (E) hind leg in dorsal view; (F) male abdominal segment VIII in ventral view; (G) pygophore in dorsal view; (H) male proctiger in dorsal view; (I) parameres in lateral view; (J) paratype labels. (K) Dorsal habitus of macropterous female from Luzon (NMNH), inset shows part of foreleg, dashed line indicates length of tibial grasping comb. Scale bar = 1.00 mm and applies to Fig A.

**Fig 22:**
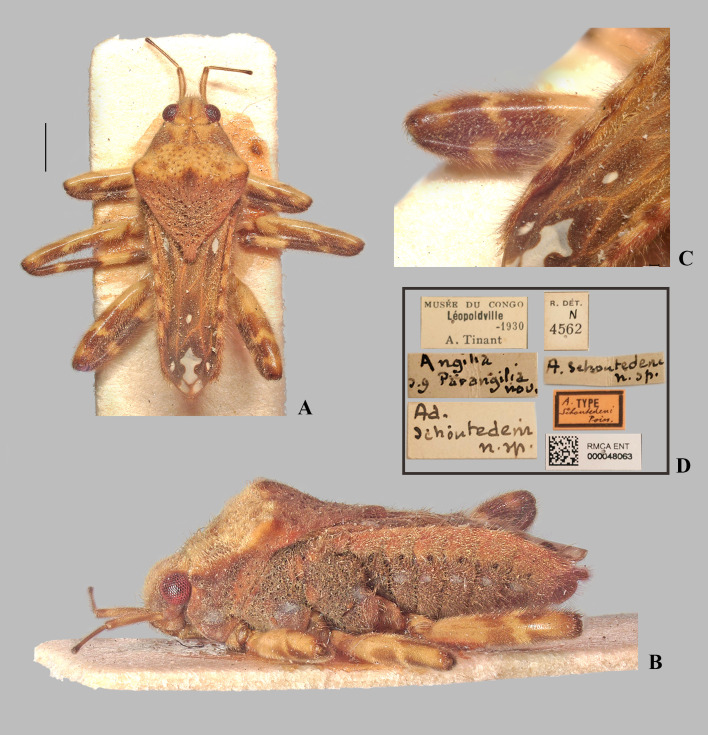
*Angilia* (*Adriennella*) *schoutedeni schoutedeni* Poisson, 1942. (A) dorsal and (B) lateral habitus of macropterous female lectotype (RMCA); (C) hind femur in dorsal view; (D) lectotype labels. Scale bar = 1.00 mm and applies to Fig A.

**Fig 23:**
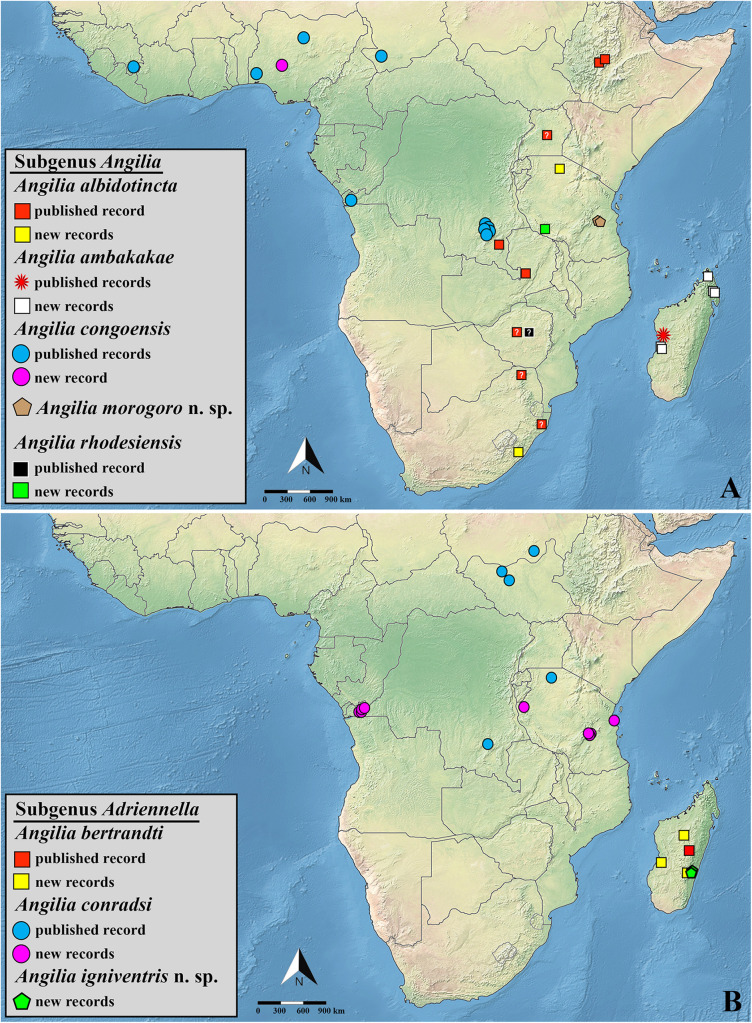
(A–B) Distribution records for species of *Angilia* in sub-Saharan Africa. Imprecise locality (e.g., only the country is known) is displayed as a question mark. All shapefiles are under open access license and free to use, credit to: Natural Earth (http://www.naturalearthdata.com/).

**Fig 24:**
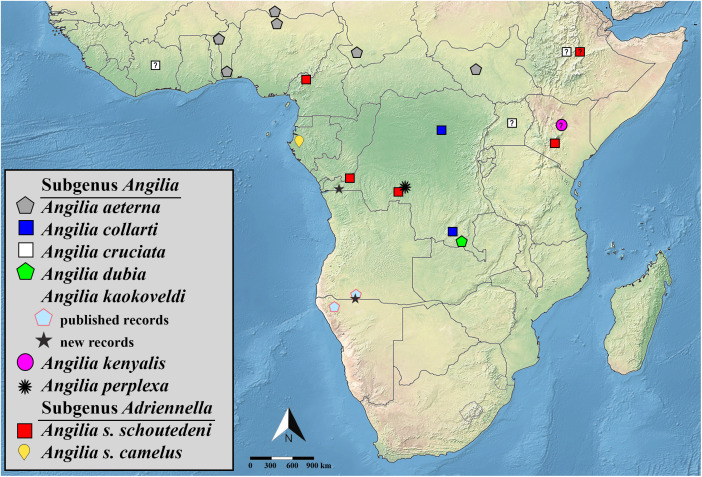
Distribution records for species of *Angilia* in sub-Saharan Africa. Imprecise localities (e.g., only the country or state is known) are displayed as a question marks. All shapefiles are under open access license and free to use, credit to: Natural Earth (http://www.naturalearthdata.com/).

**Fig 25:**
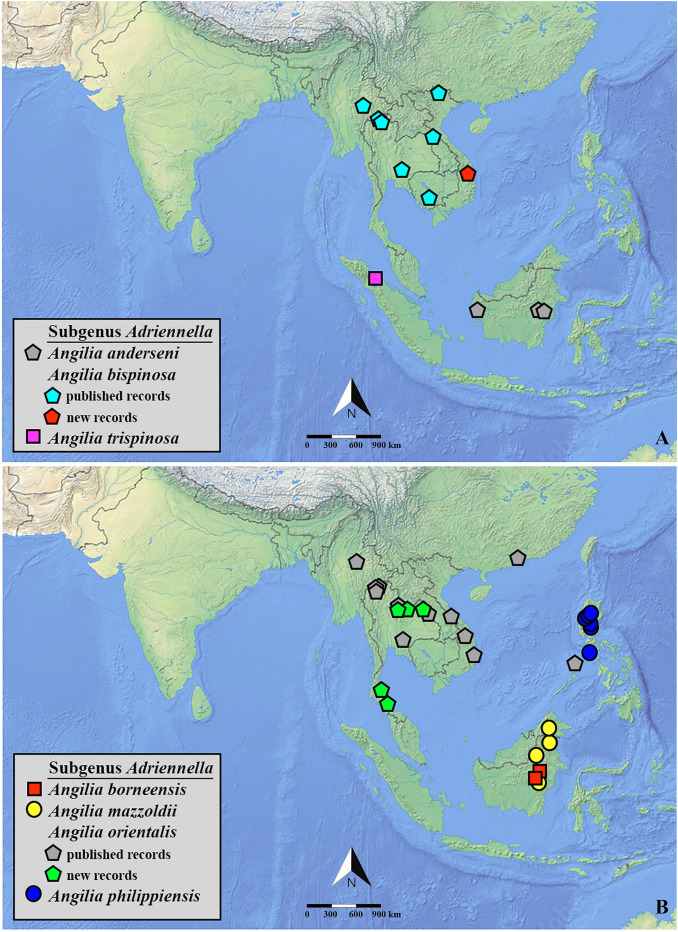
(A–B) Distribution records for species of *Angilia* in Southeast Asia. All shapefiles are under open access license and free to use, credit to: Natural Earth (http://www.naturalearthdata.com/).

*Angilia* (*Adriennella*) *anderseni* Zettel & Hecher, 1998: 338–339 (original description).

**Diagnosis.** This species can be distinguished from congeners in the subgenus by the spinose pronotal humeri (Fig 12A); the pronotum without spine or acute projection centrally; male abdominal segment VIII with a pair of bifid projections ventrolaterally (Figs 14A–B); and the body length ranges from 4.50 to 4.75.

**Discussion.** Zettel & Hecher [3] described this species from specimens collected on the island of Borneo, Indonesia. Subsequently, Zettel & Yang [11] presented a new record from the same island. Within the *A. bispinosa* group *sensu* Zettel & Hecher [3], *A. anderseni* is morphologically similar to *A. bispinosa*, mainly due to the spinose humeral angles, the absence of a spine or acute projection on the pronotal disc, and the slender and falciform paramere. Nevertheless, these species can be distinguished because in *A. anderseni*, the humeral angles are directed posterolaterally (Fig 12A), male abdominal segment VIII has a pair of bifid projections ventrolaterally (Fig 14A–B), and body length ranges from 4.50 to 4.75; whereas in *A. bispinosa*, the humeral angles are directed laterally (Figs 12D, G, 13), male abdominal segment VIII has no ventrolateral projections (Figs 14F–H), and body length ranges from 6.80 to 7.20. These two species have an allopatric distribution, with *A. anderseni* restricted to Borneo and *A. bispinosa* distributed in mainland Southeast Asia (Fig 25A).

**Distribution.** Indonesia: Kalimantan Barat [11], Kalimantan Timur [3, present study] (Fig 25A).

**Type material examined.** PARATYPES: INDONESIA, Borneo, Kalimantan Timur Prov., waterfall 4 km S. of Kota Bangun [−0.32, 116.70], VIII-29–85, CL-2095, J.T. & D.A. Polhemus/ *Angilia* (*Adriennella*) *anderseni* n. sp. Det. J.T. Polhemus/ Paratypus *Angilia anderseni* sp. n., des. Zettel & Hecher ’97/ J.T. Polhemus Collection 2014 C.J. Drake Accession/ *Angilia* (*Adr*.) *anderseni* ♂ genitalia (2♂, 1♀ macropterous NMNH).

#### *Angilia* (*Adriennella*) *bertrandi* Poisson, 1963

(Figs 15, 19A–F, 23B)

*Angilia* (*Adriennella*) *bertrandi* Poisson, 1963: 1200–1202 (original description).

**Diagnosis** This species can be distinguished from congeners in the subgenus by antennomeres III and IV slender and filiform; the pronotal humeri not spinose; the pronotum without a spine or acute projection centrally (Fig 15A); the male hind trochanter covered with small black denticles, almost absent the in female; the male hind femur with an irregular row of small black denticles, mainly on the proximal half, without distinct spines on the distal half (Fig 15B); the female hind femur without an irregular row of spines; male abdominal segment VIII without projections ventrolaterally (Figs 19A–B); and the paramere with lateral margins almost parallel and the apex subacute (Fig 18E).

**Discussion.** Only three species of *Angilia* are known from Madagascar. *Angilia bertrandi* can be easily distinguished from the other two by the absence of projections on male abdominal segment VIII (Figs 19A–B). Also, the shape of the paramere of this species (Fig 19E) is clearly different from that of *A. ambakakae* (Figs 11D–E) and A*. igniventris* **n. sp.** (Figs 19Q–S).

**Distribution.** Madagascar: Betsiboka, Haute Matsiatra, Menabe [present study], Vakinankaratra [27] (Fig 23B).

**Material examined.** MADAGASCAR, **Betsiboka**: Majunga Prov., Mamokomita Riv. and trib. 19 km SE of Andrioa, 655 m [−17.73, 47.00], XI-8–86, CL-2270, J.T. & D.A. Polhemus/ *Angilia bertrandi* Poisson Det. J.T. Polhemus/ J.T. Polhemus Collection 2014 C.J. Drake Accession (4♂, 4♀ macropterous NMNH). **Fianarantsoa Prov. [Haute Matsiatra]**: Ranomafana National Park, 3 November 2014, L-1840, R.W. Sites & S. Holmgren/ 21.23958°S, 47.37926°E, 1141 m, forest pond with sedges and lilypads (1♂ macropterous UMC). **Menabe**: Tulear Prov., river 10 km N. of Betsimba, drill site, 105 km SE of Morondava, 107 m [−20.8239, 44.4144], XI-25–86, CL-2287, J.T. & D.A. Polhemus/ *Angilia bertrandi* Poisson Det. J.T. Polhemus/ J.T. Polhemus Collection 2014 C.J. Drake Accession (3♂, 4♀ macropterous NMNH).

#### *Angilia* (*Adriennella*) *bispinosa* Andersen, 1981

(Figs 12D–K, 13, 14F–L, 25A)

*Angilia* (*Adriennella*) *bispinosa* Andersen, 1981: 345–350 (original description).

**Diagnosis.** This species can be distinguished from congeners in the subgenus by the pronotal humeri spinose (Figs 12D, G, 13); the pronotum without a spine or acute projection centrally (Fig 13); male abdominal segment VIII without projections ventrolaterally (Figs 14F–H); and the body length ranging from 6.80 to 7.20.

**Discussion.** This species is morphologically similar to *A. anderseni*, as both share spinose humeral angles, the absence of a sharp projection on the pronotum disc, and the two most distal posterolateral maculae on the forewing fused with the posteromedial macula. Nevertheless, these species can be distinguished because in *A. bispinosa*, the humeral angles are directed laterally (Figs 12D, G), male abdominal segment VIII has no ventrolateral projections (Figs 14F–H), and body length ranges from 6.80 to 7.20; whereas in *A. anderseni,* the humeral angles are directed posterolaterally (Fig 12A), male abdominal segment VIII has a pair of bifid projections (Figs 14A–B), and body length ranges from 4.50 to 4.75. *Angilia bispinosa* is the only species of the *A. bispinosa* group that occurs on mainland Southeast Asia, whereas the other three (*A. anderseni*, *A. borneensis* Zettel & Hecher, 1998, and *A. trispinosa* Andersen, 1981) occur in the Indo-Malayan Islands (Figs 25A–B).

**Distribution.** Cambodia: Kampong Speu [28]. Laos: Khammouan [11]. Myanmar: Shan [29]. Thailand: Chiang Mai [2, 29, present study], Nakhon Ratchasima [30]. Vietnam: Bắc Kạn [30], Bình Định, Gia Lai [present study] (Fig 25A).

**Type material examined.** PARATYPE: THAILAND, [**Chiang Mai**], 7 km NW of Fang. Horticultural Experimental Station [19.72, 99.08], 30.x-2.xi.1979, Zool. Mus. Copenhagen Exped./ *Angilia* (*Adr*.) *bispinosa* Andersen ♂, Det. N. Møller Andersen 1980/ Paratype/ J.T. Polhemus Collection 2014 C.J. Drake Accession (1♂ macropterous NMNH).

**Additional material examined.** THAILAND, **Chiang Mai**: trib. to Nam Chai River, Fang Horticultural Station [19.70, 99.11], CL-2202, XI-15–85, 500m, J.T. & D.A. Polhemus/ *Angilia bispinosa* Andersen ♀, Det. J.T. Polhemus (1♀ macropterous NMNH). VIETNAM, **Gia Lai**: Qui Nho’n, Dèo An Khê, along QL19 road, Gia Lai Province, 14.1585, 108.6390, Sep 2022, A. Khila, A. Badiane, Quang Ngo col. (1♂, 1♀ macropterous CEIOC); Qui Nho’n, Dèo An Khê, along QL19 road, Gia Lai [Bình Định] Province, 13.9647, 108.7633, Sep 2022, A. Khila, A. Badiane, Quang Ngo col. (3♂, 1♀ macropterous CEIOC).

#### *Angilia* (*Adriennella*) *borneensis* Zettel & Hecher, 1998

*Angilia* (*Adriennella*) *borneensis* Zettel & Hecher, 1998: 339–340 (original description).

**Distribution.** Indonesia: Kalimantan Timur [3,11] (Fig 25B).

#### *Angilia* (*Adriennella*) *conradsi* Poisson, 1950

(Figs 16, 17, 19G–L, 23B)

*Angilia* (*Adriennella*) *conradsi* Poisson, 1950: 83–84 (original description).

**Diagnosis.** This species can be distinguished from congeners in the subgenus by antennomeres III and IV slender and filiform; the pronotal humeri not spinose; the pronotum without a spine or acute projection centrally (Figs 16A, C, 17A, E); the male hind trochanter with a few small black denticles posteriorly (Fig 15D), almost absent in the female (Fig 17F); the male hind femur with black denticles scattered on the anterior half, almost absent in the female; male abdominal sternum VII with a rounded lobe extended distally at the posterolateral corner (Figs 16E, 17C); male abdominal segment VIII with a pair of lateral projections at mid-length, curved distally (Figs 19G–I); and the paramere with shallowly sinuate lateral margins and apex broadly rounded (Figs 19J–K).

**Lectotype designation.** Poisson [5] described this species based on a male and a female, both macropterous, collected in Ukerewe Island, southeastern Lake Victoria, Tanzania. He indicated that these specimens were deposited in the “Musée du Congo”, now known as the RMCA. Poisson did not designate a holotype in the original description, nor in subsequent papers. However, the male is labeled “Holotypus” (Fig 16F) and the female, “Paratypus”. The mere addition of these labels, which was probably not done by Poisson himself, does not formalize the type designation. Therefore, to ensure nomenclatural stability, the male is formally designated here as the lectotype and the female as the paralectotype.

**Discussion.** The lectotype is in relatively good condition, despite the absence of two legs (Fig 16). Abdominal segment VIII and the genital capsule have been dissected, but these structures are not attached to the specimen (they are probably in Poisson’s slide collection, purchased by the NMNH). The posterolateral angles of abdominal sternum VII are weakly rounded lobes, a feature that exhibits variation among the additional specimens examined, some of which have the rounded lobes more prominent.

This species is morphologically similar to *A. schoutedeni schoutedeni* Poisson, 1942. Both taxa share the same color pattern and general body morphology, but can be distinguished by the shape of the pronotum, the male hind leg and abdominal segment VIII. In *A. conradsi*, the pronotum is only slightly elevated between the humeral angles (Figs 16C, 17D), the hind femur has only a few scattered black denticles on the proximal half (Figs 16D, 17B, F), and the lateral projection of abdominal segment VIII is curved distally (Figs 19G–I); whereas in *A. s. schoutedeni,* the pronotum is distinctly elevated between the humeral angles (Fig 22B), the hind femur has black denticles along the entire posterior margin, and the lateral projection of abdominal segment VIII is straight distally.

We observed variation in the shape of the paramere, which in some specimens has the posterodorsal angle acute, very similar to the paramere of *A. s. schoutedeni* illustrated by Poisson ( [5]: 85, Fig 18d). However, the same author had already provided illustrations in different views of the paramere of *A. conradsi* (see Poisson [25]: 7, Fig 2e), with the posterodorsal angle acute as in *A. s. schoutedeni*, showing a similar shape between these species.

**Distribution.** Democratic Republic of the Congo: Kongo Central [present study], Haut-Lomami [25]. South Sudan: Northern Bahr el Ghazal, Upper Nile, Warrap [18]. Tanzania: Kigoma [present study], Mwanza [5], Morogoro, Unguja North [present study] (Fig 23B).

**Type material examined.** LECTOTYPE ♂ [herein designated] macropterous (RMCA), [TANZANIA], Coll. Mus. Congo, Victoria Nyanza, Ukerewe [−2.03, 33.03], VII-1938, R.P. Conrads, 371/ R. DET. T. 5493/ *Angilia conradsi* n. sp./ Holotypus/ RMCA ENT 000048055.

**Additional material examined.** DEMOCRATIC REPUBLIC OF THE CONGO: [**Kongo Central]:** Bas Congo Province: Sanzikwa River, 7 August 2012, L-1443, Sites, Shepard, Pwema/ 309 m, 05°38.542’S, 14°13.037’E, vegetated margins of gravel stream (2♀ macropterous UMC); Bas Congo Province, Fwadiagombe River, ca. 4 km SW of Snell-Kwilu power station, 7 August 2012, L-1444/ Sites, Shepard & Pwema/ 05°37.689’S, 14°14.368’E, 329 m, vegetated margins of gravel stream (1♂ macropterous UMC); Bas Congo Province, Lukusu River nr Mboma, 11 km S of Mayidi, 8 August 2012, L-1448, Sites, Shepard & Pwema/ 05°13.615’S, 14°13.042’E, 537 m, vegetated margins of slow stream (1♂ macropterous UMC); Bas Congo Province, Lukusu River, 7 km N of Madimba, vegtd. margins of stream, 9 August 2012, L-1452/ 467 m, 04°56.288’S, 15°08.863’E, colls: Sites, Shepard & Pwema (1♂ macropterous UMC). TANZANIA, **Kigoma**: Kibaoni Stream, 30 July 2010, L-1183, R.W. Sites & A. Mbogho, pooled areas of slow stream, 05°03.974’S, 30°20.666’E, 1026 m (2♂ macropterous UMC). **Morogoro**: Kilombero District, Mwaya River at Mangula, 7 August 2013, L-1623, Sites, Mbogho, Rwegasira/ 313 m, 07°50.945’S, 36°53.201’E, muddy, vegetated margin of sand, gravel stream (4♂, 4♀ macropterous UMC); Kisawasawa, River at Kisawasawa, 8 August 2013, L-1625, Sites, Mbogho, Rwegasira/ 326 m, 07˚53.596’S, 36˚52.311’E, vegetated margins of sandy stream (4♂, 1♀ macropterous UMC; 1♂, 1♀ macropterous CEIOC); Sonjo River at Sonjo, 8 August 2013, colls: Sites, Mbogho & Rwegasira, L-1626/ 320 m, 07˚48.490’S, 36˚53.785’E, vegetated margins of rocky stream (8♂, 8♀ macropterous UMC). **[Unguja North]**: Zanzibar, Kaskazini Region, Kitope River at Zingwezingwe Bridge, 17 August 2010, L-1265/ discontinuous pools with aquatic vegetation & grasses, 06°00.963’S, 39°14.734’E, 39 m, R. Sites & A. Mbogho (1♀ macropterous UMC; 1♀ macropterous CEIOC); Zanzibar, Kaskazini Region, Kitope River at Mkaratini Bridge, 17 August 2010, L-1266/ discontinuous pools with aquatic vegetation & grasses, 06°00.714’S, 39°14.841’E, 30 m, R. Sites & A. Mbogho (1♂, 2♀ macropterous UMC; 1 macropterous CEIOC).

#### *Angilia* (*Adriennella*) *igniventris* Rodrigues, Sites & Moreira n. sp.

(Figs 18, 19M–S, 23B)

urn:lsid:zoobank.org:act:78CDFDA8-7541-4CDC-A149-B3483DE36A28

**Description.** Macropterous male: HOLOTYPE (Fig 18), length 6.16; maximum width 2.28. PARATYPE (n = 1), length 5.92, maximum width 2.20.

*Color* General dorsal coloration brown, with yellow-banded legs; ventrally, dark-brown on thoracic sterna, orange-red on abdominal sterna. Antenna light-brown. Labium with articles I–III brown to light-brown, IV black. Forewing dark-brown, veins pale-brown; a sub-ovate white macula at base, not exceeding posterior margin of pronotum; a small white macula at mid-length of posterior discal cell; and one large, irregularly shaped macula and four smaller maculae at distal part of wing (Fig 18A). Legs with coxae and trochanters yellowish-brown; femora, tibiae, and tarsomeres brown dorsally, lighter ventrally; each femur with a faint band at middle; each tibia with two yellow bands, one at basal third and another at apex. Abdominal sterna with a longitudinal, orange-brown band laterally.

*Pilosity*. Head, antenna, pronotum, legs, lateral edge of abdominal laterotergites, pleura, and abdominal sterna covered by fine golden pubescence intermixed with elongate dark-brown setae. Anterior lobe of pronotum with faint, small, frosty pubescence posterolaterally. Forewing with elongate dark-brown setae on basal veins.

*Head.* Short, strongly deflected in front of eyes, with impressed median line; pair of narrow indentations near mesal margins of eyes; length 0.68, width including eyes 1.28; eye width 0.33. Ocular setae present. Antenniferous tubercle wide and swollen. Antennomere I thicker than others, curved laterally; II slightly thicker than III and IV; III and IV subequal in width; IV filiform; length of antennomeres I–IV, 1.04, 0.88, 1.08, 1.00. Labium reaching middle of mesosternum.

*Thorax.* Pronotum length along midline 2.80, maximum width 2.28; humeri raised; posterior lobe covered by rounded punctations, with a low carina along longitudinal midline, and a finger-like projection medially at distal tip. Forewing reaching tip of abdomen, leaving only posterior portion of genital capsule exposed, with four closed cells. Proepimeron with a row of rounded punctations. Mesepimeron with scattered rounded punctations. Each acetabulum with faint, frosty pubescence. Meso- and metasterna centrally without two pairs of small tubercles on intersegmental region. Metasternum with posterior margin convex.

*Legs*. Foretibia with grasping comb (1.34 long) occupying 3/4 its length. Middle tibia with a row of elongate dark-brown trichobothria-like setae on distal half, decreasing in size distally. Hind trochanter bearing small, dark-brown teeth or pegs ventrally. Hind femur distinctly thicker than others, slightly dilated at mid-length, bearing irregular rows of small, dark-brown teeth or pegs ventrally. Hind tibia with scattered, dark-brown pegs ventrally, mainly on posterior third. Leg measurements as follows: foreleg, femur 1.80, tibia 1.84, tarsomeres 1–3, 0.10, 0.20, 0.50; middle leg, femur 2.48, tibia 1.20, tarsomeres 1–3, 0.20, 0.74, 0.76; hind leg, femur 2.72, tibia 2.44, tarsomeres 1–3, 0.18, 0.66, 0.66.

*Abdomen.* Lateral margins subparallel. Laterotergites not elevated, without black denticles. Laterally, on region of insertion of lateral abdominal muscles, with a pair of sub-ovate furrows on sterna II–VII. Sterna IV–V with longitudinal areas with few setae at midline. Sternum VII with posterolateral corners forming small, rounded lobes (Fig 18B). Segment VIII widened on posterior half dorsally, with two pairs of projections, one smaller and directed ventrally, and another larger, anterolaterally oriented, covered by golden setae (Figs 19M–P). Proctiger with golden setae posteriorly. Paramere curved, widened at mid-length, with a notch posteroventrally, and distinctly tapered posteriorly (Figs 19Q–S).

### Macropterous female. Unknown

**Distribution and habitat.** This species is known from two localities in Ranomafana National Park, southeastern Madagascar (Fig 23B). The holotype was collected in a forest pond with sedges and lily pads, and the paratype in a shallow, muddy, mossy forest pool that also harbored many individuals of *Laccotrephes* (Nepidae).

**Diagnosis.** This species can be distinguished from all congeners by the unique shape of the paramere (Figs 19Q–S). In addition to this structure, it also differs from the others by the orange-red abdomen contrasting to the rest of the brown body (Fig 18) and in the shape of male abdominal segment VIII, which has two pairs of projections, one smaller and directed ventrally, and the other larger, anterolaterally directed, covered with golden setae (Figs 19M–P).

**Discussion.** This species belongs to the subgenus *A*. (*Adriennella*), because it has a finger-like projection medially on the posterior margin of the pronotum and the middle tarsus is about 2/3 the length of the middle tibia. In Madagascar, two species have been recorded so far, one in each subgenus: *A.* (*Angilia*) *ambakakae* and *A*. (*Adriennella*) *bertrandi*. This third, new species is easily distinguished from the congeners by the distinct shape of the paramere, which is unique among all congeners. The uniform brown color of the pronotum, the orange color of the abdominal sterna, the basal macula of the forewing not exceeding the posterior margin of the pronotum, and male abdominal segment VIII with two pairs of ventral projections are also diagnostic.

**Etymology.** The specific epithet derives from Latin, *ignis* (fire) and *ventris* (belly), and refers to the color of the abdominal sterna.

**Type material examined.** HOLOTYPE, ♂ macropterous (UMC), MADAGASCAR, [**Haute Matsiatra]**: Fianarantsoa Prov., Ranomafana National Park, 3 November 2014, L-1840, R.W. Sites & S. Holmgren/ 21.23958°S, 47.37926°E, 1141 m, forest pond with sedges and lilypads. PARATYPE: Ranomafana National Park, 1138 m, 21°14.259’S, 47°23.786’E, 2 November 2014, L-1832/ R.W. Sites, J. Bergsten & S. Holmgren, shallow muddy, mossy forest pool (1♂ macropterous UMC).

#### *Angilia* (*Adriennella*) *mazzoldii* Zettel & Hecher, 1998

*Angilia* (*Adriennella*) *mazzoldii* Zettel & Hecher, 1998: 336–338 (original description).

**Distribution.** Indonesia: Kalimantan Timur [3,11]. Malaysia: Sabah [11] (Fig 25B).

#### *Angilia* (*Adriennella*) *orientalis* Andersen, 1981

(Figs 14M–P, 20, 25B)

*Angilia* (*Adriennella*) *orientalis* Andersen, 1981: 343–345 (original description).

**Diagnosis.** This species can be distinguished from congeners in the subgenus by the pronotal humeri not spinose; the pronotum disc not highly elevated between the humeral angles (Figs 20A, E); the male hind trochanter with small black denticles posteriorly, almost absent in females; the hind femur with an irregular row of small black denticles on the posterior margin in both sexes; the female grasping comb longer than half the length of the foretibia; male abdominal segment VIII without projections (Figs 14M–O); and the paramere slightly widened at mid-length, with the apex broadly rounded (Fig 14P).

**Discussion.** This species is morphologically similar to *A. philippiensis* Drake & Hoberlandt, 1953 and both share the absence of spines on the humeral angles, a pronotal disc without an acute projection or elevation, and male abdominal segment VIII without an acute processes ventrolaterally. Nevertheless, these species can be distinguished because in *A. orientalis*, male abdominal segment VIII is not ventrolaterally excavated (Fig 14N), the paramere has a rounded apex (Fig 14P), and the female grasping comb is longer than half of the foretibia (Fig 20F); whereas in *A. philippiensis*, male abdominal segment VIII is ventrolaterally excavated at mid-length (Fig 21F), the paramere has a truncated apex (Fig 21I), and the female grasping comb is shorter than half the length of the foretibia. *Angilia orientalis* is the only species of the *A. orientalis* group that occurs in mainland Southeast Asia, whereas the other two, *A. mazzoldii* Zettel & Hecher, 1998 and *A. philippiensis*, occur in the Indo-Malayan Islands. Zettel & Hecher [3] examined three males of *A. orientalis* from the Philippines and reported no significant differences with specimens from Thailand. Furthermore, the authors suggested that the Mindoro Strait may represent the geographic limit of the distribution of these two species (Fig 25B).

**Distribution.** China: Hong Kong [2]. Myanmar: Mandalay [29, present study]. Philippines: Palawan [3]. Thailand: Chiang Mai [2,3,11,29, present study], Loei [11, present study], Mukdahan [11], Phetchabun [present study], Phrae [3], Prachinburi [2], Sakon Nakhon, Songkhla, Surat Thani [present study]. Vietnam: Kon Tum, Phú Yên [30] (Fig 25B).

**Material examined.** MYANMAR, **Mandalay**: Burma, Mandalay Division, spring outflows nr. Nyaungkhangyi, 7 km N.of Maymyo, 1065 m, 20 Oct 1998, D.A. & J.T. Polhemus, CL-4013, 22°05.37’N, 96°27.41’E/ *Angilia orientalis* Andersen, Det. J.T. Polhemus/ J.T. Polhemus Collection 2014 C.J. Drake Accession (1♂, 1♀ macropterous NMNH). THAILAND, **Chiang Mai**: trib. to Nam Chai River, Fang Horticultural Station [19.70, 99.11], CL-2202, XI-15–85, 500 m, J.T. & D.A. Polhemus/ *Angilia orientalis* Andersen, Det. J.T. Polhemus/ J.T. Polhemus Collection 2014 C.J. Drake Accession (1♀ macropterous NMNH); Mae Sa Waterfall [18.906, 98.897], 7 km W. of Mae Rim, CL-2203, XI-18–85, J.T. & D.A. Polhemus/ *Angilia orientalis* Andersen, Det. J.T. Polhemus/ J.T. Polhemus Collection 2014 C.J. Drake Accession (1♂ macropterous NMNH). **Loei**: 3.0 km N Nong Kang [17.4575, 101.4142], 11 June 1998, L-179, Sites, Simpson, Vitheepradit, veg. pond nr rice paddy (1♂ macropterous UMC); Ban Nong Ngyu [17.4575, 101.4142], 11 June 1998, L-183, Sites, Simpson, Vitheepradit, unplanted rice paddy (1♂ macropterous UMC). **Phetchabun**: Amphur Lom Sak, Ban Na Kham [16.7736, 101.2461], 20 June 1998, L-198, colls: Vitheepradit & Sawangsak, vegetated pond (1♂ macropterous UMC). **Sakon Nakhon**: Huay Huad N. P., Huay Nam Sai [16.9827, 104.0694], 6 June 1998, L-158, Sites, Simpson, Vitheepradit, narrow creek with vegetation/ *Angilia orientalis* Andersen, det RWS 1999. (1♀ macropterous UMC). **Songkhla**, PSU [Prince of Songkla University] Campus [7.01, 100.50], Hat Yai, 8 January 1995, L-67, colls: B. Nichols & R. Sites, vegetated ponds/ L-67, *Angilia orientalis* (1♀ macropterous UMC). **Surat Thani**: Amphur Phunphin, Tumbon Boh Rai, 7 June 2004, L-751, Vitheepradit & Prommi/ pond, 10 m, 08°53.866’N, 99°08.961’E (1♀ macropterous UMC).

#### *Angilia* (*Adriennella*) *philippiensis* Drake & Hoberlandt, 1953.

(Figs 21, 25B)

*Angilia* (*Adriennella*) *philippiensis* Drake & Hoberlandt, 1953: 223–226 (original description).

**Diagnosis.** This species can be distinguished from congeners in the subgenus by the pronotal humeri not spinose (Figs 21A, K); the pronotum disc not highly elevated between the humeral angles, with shorter setae; the female grasping comb shorter than half the length of the foretibia; male abdominal segment VIII excavated ventrolaterally at mid-length, without projections or process (Fig 21F); and the paramere slightly narrowed at mid-length, with the apex truncated (Fig 21I).

**Discussion.** This species is morphologically similar to *A. orientalis*. For a comparison, see discussion under the previous species.

**Distribution.** Philippines: Kanlurang Mindoro [31, present study], La Union [2], Mountain [11,32], Nueva Ecija [11,29, present study], Rizal [2] (Fig 25B).

**Type material examined.** PARATYPE: [PHILIPPINES, **Kanlurang Mindoro**], San Jose [12.3, 121.1], Mindoro, P.I., IV-45, E.S. Ross/ *Angilia philippiensis* D & H, paratye/ Paratypus *Angilia* (*Adriennella*) *philippiensis* sp. n., C.J. Drake et L. Hoberlandt, 1952 ♂/ Paratypus/ CJ Drake Coll. 1956 (1♂ macropterous NMNH).

**Additional material examined.** PHILIPPINES, **Nueva Ecija**: Luzon, N. Ecija, Carranglan, Maringalo [15.96, 121.02], BFD Station, creek, 5 Nov. 1976, A.A. Barroso/ *Angilia* (*Adriennella*) *philippiensis* Dr & Hob. ♀, Det. J.T. Polhemus/ J.T. Polhemus Collection 2014 C.J. Drake Accession (1♀ macropterous NMNH).

#### *Angilia* (*Adriennella*) *schoutedeni schoutedeni* Poisson, 1942

(Figs 22, 24)

*Angilia schoutedeni* Poisson, 1942: 161–164 (original description).

*Angilia* (*Adriennella*) *schoutedeni*: Poisson 1955: 278 (subgeneric placement).

**Diagnosis.** This species can be distinguished from congeners in the subgenus by the distinct elevation between the humeral angles of pronotum, but without forming a spine or sharp projection; and the pronotal humeri not spinose (Figs 22A–B).

**Lectotype designation.** Poisson [4] described this species based on two females from the Democratic Republic of the Congo, one collected in Léopoldville (= currently Kinshasa) and the other in Luebo. These two females, one of which was examined here, are deposited at the RMCA (Fig 22). Poisson did not designate the holotype in the original description, nor the lectotype in subsequent publications. Therefore, to ensure nomenclatural stability, the female from Kinshasa is formally designated here as the lectotype and the other female as the paralectotype.

**Discussion.** The lectotype examined has its ventral surface glued to a paper card, which makes it impossible to view some of the structures. Antennomeres III and IV are missing and the position of the hind femora and tibiae also prevents the visualization of the posterior margin of these structures.

After describing *A. schoutedeni*, Poisson [5] proposed the subspecies *A. schoutedeni camelus*, based on a female from Lambaréné, the capital of Moyen-Ogooué Province, Gabon. Poisson distinguished *A. s. camelus* from the nominal subspecies by the more pronounced elevation between the humeral angles of the pronotum and by the more elongate antennomeres. In the original description, Poisson [4] indicated that the holotype female of *A. s. camelus* was deposited in his personal collection; however, we were unable to locate this specimen. The separation of these two subspecies is based on features that can vary, such as the height of the pronotum, which raises the possibility that they are synonyms. However, because it was not possible to examine the type of *A. s. camelus*, we do not propose any taxonomic changes at this time.

This species is morphologically similar to *A. conradsi*. For a comparison, see discussion under that species.

**Distribution.** Cameroon: l’Ouest [33]. Democratic Republic of the Congo: Kasaï [4], Kinshasa [4, present study]. Ethiopia: Omo River [34]. Kenya: Kiambu [35] (Fig 24).

**Type material examined.** LECTOTYPE [herein designated] ♀ macropterous (RMCA), [DEMOCRATIC REPUBLIC OF THE CONGO], Musée du Congo, Léopoldville [−4.3, 15.3], 1930, A. Tinant/ R. Dét. N 4562/ *Angilia Parangilia* nov./ *A. schoutedeni* n. sp./ *Ad. schoutedeni* n. sp./ TYPE *A. schoutedeni* Poiss./ RMCA ENT 000048063.

#### *Angilia* (*Adriennella*) *schoutedeni camelus* Poisson, 1950

*Angilia* (*Adriennella*) *schoutedeni camelus* Poisson, 1950: 84–85 (original description).

**Distribution.** Gabon: Moyen-Ogooué [5] (Fig 24).

**Discussion.** This subspecies was proposed based on a single macropterous female from Gabon (Poisson [5]). This record falls within the known distribution range of the nominate subspecies. According to the author, this taxon may be a species, but this hypothesis can only be confirmed through male specimens. Poisson [5] stated that the holotype was originally deposited in his collection. After his death, the collection was fragmented and acquired by different institutions.

#### *Angilia* (*Adriennella*) *trispinosa* Andersen, 1981

*Angilia* (*Adriennella*) trispinosa Andersen, 1981: 350–351 (original description).

**Distribution.** Indonesia: Sumatera Utara [2,29] (Fig 25A).

#### Key to subgenera and species of *Angilia.*

In part modified from Zettel & Hecher [3].

**Notes.** This key is primarily intended for the identification of males. Some species could not be examined directly. In these cases, the diagnostic characters were based exclusively on the literature. Since no species has been recorded in more than one of the three regions – mainland Africa, Madagascar, and Southeast Asia –, geographic distribution was used as an additional criterion for species identification. Also, *A. dubia* and *A. perplexa* were not included in the key because males are unkown; see Diagnosis section for these species.

1. Pronotum without a finger-like projection at posterior angle (Figs 1A, 2A); female foretibial grasping comb distinctly shorter than that of male, usually less than 1/3 (Figs 2B, 3B); middle tarsus subequal in length to middle tibia (Fig 2B).....***Angilia* (*Angilia*)**.....2
2. Pronotum with a finger-like projection at posterior angle (Figs 12A, D, G); grasping comb of female foretibia almost as long as that of male (Figs 12H–I); middle tarsus about 2/3 length of middle tibia (Fig 12D, G).....***Angilia* (*Adriennella*)**.....11
3. Geographic distribution: Madagascar.....***Angilia ambakakae***
4. Geographic distribution: maindland Africa.....3
5. Male abdominal segment VIII lacking projections (Figs 11F–H).....4
6. Male abdominal segment VIII with lateral and ventrolateral projections (Figs 11L–N).....7
7. Hind femur, in both sexes, with one large ventral spine located slightly beyond the middle of segment, in addition to irregular rows of small, dark-brown spines (Figs 2B, 3B, 8B, E).....5
8. Hind femur, in both sexes, only with irregular rows of small, dark-brown spines, without a large spine (Figs 7A–D).....***Angilia kaokoveldi***
9. Forewing basal macula originating from beyond humeral angle (Figs 8A, D); paramere apex truncate (Figs 11J–K).....***Angilia morogoro* n. sp.**
10. Forewing basal macula originating from humeral angle (Figs 1A, 2A, 3A); paramere apex acute (Poisson [7]: Figs 7e–f; Hoberlandt [16]: Figs 2d–e).....6
11. Forewing lacking mid-lateral macula (Fig. 1A)…..***Angilia aeterna***
12. Forewing with mid-lateral macula (Figs 2A, 3A).....***Angilia albidotincta***
13. Lateral and ventrolateral projections of abdominal segment VIII of similar lengths (Poisson [8]: Figs 10c–d).....8
14. Lateral projection of abdominal segment VIII at least twice as long as ventrolateral projection (Fig 11M)…..9
15. Projections of male abdominal segment VIII elongated, length greater than 1.5× width (Poisson [8]: Fig 10d).....***Angilia collarti***
16. Projections of male abdominal segment VIII subrounded, length only slightly greater than width (Poisson [8]: Fig 10c).....***Angilia kenyalis***
17. Hind femur, in both sexes, with one or two large ventral spines located slightly beyond the middle of segment, in addition to irregular rows of small, dark-brown spines (Figs 9C, 10B).....10
18. Hind femur, in both sexes, only with irregular rows of small, dark-brown spines, without large spines (Fig. 6A).....***Angilia congoensis***
19. Forewing basal macula extending to beyond posterior margin of pronotum (Poisson [7]); paramere with anterodorsal margin distinctly notched and apical third distinctly tapered (Poisson [7])….. ***Angilia cruciata***
20. Forewing basal macula usually ending at same level of posterior margin of pronotum (Figs 9A, 10A); paramere poorly notched anterodorsally, with apical third evenly tapered (Fig 11O).....***Angilia rhodesiensis***
21. Geographic distribution: mainland Africa or Madagascar.....12
22. Geographic distribution: Southeast Asia.....16
23. Geographic distribution: Madagascar.....13
24. Geographic distribution: mainland Africa.....14
25. Pronotum posterior lobe yellow anteriorly and brown posteriorly (Fig 15A); abdomen mostly brown ventrally (Figs. 15B–C); male abdominal segment VIII lacking projections (Figs 19A–C); paramere with lateral margins almost parallel and apex subacute (Fig 19E).....***Angilia bertrandi***
26. Pronotum general color brown to reddish-brown (Fig 18A); abdomen mostly orange-red ventrally (Figs 18B–C); male abdominal segment VIII with two pairs of projections (Figs 19M–P); paramere widened at mid-length, with a notch posteroventrally, and distinctly tapered posteriorly (Figs 19Q–S).....***Angilia igniventris* n. sp.**
27. Pronotum slightly elevated between humeral angles (Figs 16C, 17D); hind femur with few scattered black denticles on proximal half (Figs 16D, 17B); lateral projection of abdominal segment VIII curved distally (Figs 19G–I).....***Angilia conradsi***
28. Pronotum distinctly elevated between humeral angles (Fig 22B); hind femur with black denticles along entire posterior margin; lateral projection of abdominal segment VIII straight distally (Poisson [5]: fig 18c).....***Angilia schoutedeni***.....15
29. Antennomere II longer than IV; posterior angle of pronotum straight, not curved ventrally (Fig 22).....***Angilia schoutedeni schoutedeni***
30. Antennomere II shorter than IV; posterior angle of pronotum curved ventrally (Poisson [5]: fig 19b).....***Angilia schoutedeni camelus***
31. Pronotum humeral angles spinose (Figs 12A, D, G); paramere thin, falciform (Figs 14E, K–L)…..***Angilia bispinosa* group**…..17
32. Pronotum humeral angles not spinose; paramere broad, with apex rounded or truncate (Figs 14P, 21I).....***Angilia orientalis* group**.....20
33. Pronotum with a spine or cone-shaped projection centrally (Zettel & Hecher [3]: figs 4, 13–14).....18
34. Pronotum without spine or projection centrally (Figs 12B,E, 13).....19
35. Pronotum with a cone-shaped projection centrally; female tibial grasping comb one-fifth length of fore tibia.....***Angilia borneensis***
36. Pronotum with a spine centrally; female tibial grasping comb more than one-third length of fore tibia.....***Angilia trispinosa***
37. Pronotum humeral angles directed posterolaterally (Fig 12A); body length 4.50–4.75; male abdominal segment VIII with a pair of bifid projections (Figs 14A–B).....***Angilia anderseni***
38. Pronotum humeral angles usually directed laterally (Fig 12D, G); body length 6.80–7.20; male abdominal segment VIII without projections (Figs 14F–H).....***Angilia bispinosa***
39. Pronotum distinctly elevated centrally, with long setae; male abdominal segment VIII with sharp projections ventrolaterally (Zettel & Hecher [3]: fig 9); distribution: Borneo.....***Angilia mazzoldii***
40. Pronotum not distinctly elevated centrally, with short setae; male abdominal segment VIII without sharp projections ventrolaterally (Figs 14M–O, 21F); distribution: mainland Southeast Asia and Philippines.....21
41. Male abdominal segment VIII not excavated ventrolaterally (Fig 14N); paramere apex rounded (Fig 14P); female grasping comb longer than half length of foretibia (Fig 20F).....***Angilia orientalis***
42. Male abdominal segment VIII excavated ventrolaterally (Fig 21F); paramere apex truncated (Fig 21I); female grasping comb shorter than half length of foretibia (Fig 21K).....***Angilia philippiensis***
